# Magnetometry with a space-based differential atom interferometer

**DOI:** 10.1038/s41467-026-75230-2

**Published:** 2026-07-11

**Authors:** Matthias Meister, Gabriel Müller, Patrick Boegel, Albert Roura, Annie Pichery, David B. Reinhardt, Timothé Estrampes, Jannik Ströhle, Enno Giese, Holger Ahlers, Waldemar Herr, Christian Schubert, Éric Charron, Holger Müller, Jason R. Williams, Ernst M. Rasel, Wolfgang P. Schleich, Naceur Gaaloul, Nicholas P. Bigelow

**Affiliations:** 1 German Aerospace Center (DLR), Institute of Quantum Technologies, Ulm, Germany; 2 Institute of Quantum Optics, QUEST-Leibniz Research School, Leibniz University Hannover, Hanover, Germany; 3 Institut für Quantenphysik and Center for Integrated Quantum Science and Technology (IQST), Ulm University, Ulm, Germany; 4 Université Paris-Saclay, CNRS, Institut des Sciences Moléculaires d’Orsay, Orsay, France; 5 Institut für Angewandte Physik, Fachbereich Physik, Technische Universität Darmstadt, Darmstadt, Germany; 6 German Aerospace Center (DLR), Institute for Satellite Geodesy and Inertial Sensing, Hanover, Germany; 7 Department of Physics, University of California, Berkeley, CA USA; 8 Jet Propulsion Laboratory, California Institute of Technology, Pasadena, CA USA; 9 Hagler Institute for Advanced Study, Texas A&M University, College Station, TX USA; 10 Texas A&M AgriLife Research, Texas A&M University, College Station, TX USA; 11 Institute for Quantum Science and Engineering (IQSE), Department of Physics and Astronomy, Texas A&M University, College Station, TX USA; 12 Center for Coherence and Quantum Optics, Institute of Optics, Department of Physics and Astronomy, University of Rochester, Rochester, NY USA

**Keywords:** Matter waves and particle beams, Bose-Einstein condensates, Ultracold gases

## Abstract

Atom interferometers deployed in space are excellent tools for high precision measurements, navigation, or Earth observation. In particular, differential interferometric setups feature common-mode noise suppression and enable reliable measurements in the presence of ambient platform noise. Here we report on orbital magnetometry campaigns performed with differential single- and double-loop interferometers in NASA’s Cold Atom Lab aboard the International Space Station. By comparing measurements with atoms in magnetically sensitive and insensitive states, we have realized atomic magnetometers mapping magnetic field curvatures. Our results pave the way towards precision quantum sensing missions in space.

## Abstract (web summary)

Atom-interferometers-based quantum sensors in space promise high-precision fundamental physics tests and geophysical applications. Here, the authors demonstrate the measurement of magnetic field curvatures via differential Bose-Einstein condensate interferometers at the NASA Cold Atom Laboratory aboard the International Space Station, by suppressing the effect of vibrational noise and improving atom source control.

## Introduction

Deploying matter-wave interferometers^1^ to space^2–5^ promises the advance of fundamental physics by testing general relativity^6–10^, detecting gravitational waves^11,12^ and exploring new physics^13,14^. Their use in applied sensors^15^ could enable, for instance, long-term inertial navigation^16–19^, precision gravity cartography^20–23^ and enhanced magnetic field sensing^23–30^. For matter-wave interferometers to benefit from space and exceed the performance of classical sensors, they need to exploit extended free-fall times enabling quadratic improvements in sensitivity. These long interferometry times can best be realized by using slowly expanding Bose–Einstein condensates (BECs)^31–34^ as quantum test masses.

Until now, the application of space-deployed BEC interferometers as sensors and at extended free-fall times has remained elusive. Proof-of-principle experiments with BECs were performed using double Bragg interferometry aboard a sounding rocket^35^ and single Bragg interferometry with the Cold Atom Lab (CAL) onboard the International Space Station (ISS)^36^. Additionally, a dual-species BEC interferometer, suitable conceptually for testing general relativity, has been carried out with CAL^37^. Finally, using thermal atoms rather than BECs, a double Raman interferometry-based gyroscope was realized in the China Space Station^19^. In contrast to the latter, all space-borne BEC interferometers were limited in their sensitivity by short interrogation times, mainly due to laser phase and vibrational noise in the non-differential^36^ or insufficient control of the atom dynamics in the differential case^37^.

Overcoming previous limitations, we apply a BEC interferometer as a magnetic field sensor in the Earth-orbiting CAL experiment. To improve the sensitivity, we thoroughly characterize the interferometer beam to inform and apply advanced state-engineering techniques^34,38^ enabling longer spatial overlap of condensed ^87^Rb atoms with the interferometer beam and thus increased pulse efficiencies for long times. Additionally, using single Bragg pulses, we execute differential interferometry schemes suppressing laser phase noise in the vibrationally noisy environment of the ISS, while directly measuring second-order potential gradients (magnetic field curvature) where a non-differential scheme would measure the first order. These modifications allowed the execution of various interferometer geometries up to total interrogation times of 2*T* = 40.3 ms, significantly improving upon previous space-based realizations of BEC interferometers^35–37^ where visibilities could not be shown beyond 2*T* = 4 ms due to insufficient pulse efficiencies.

In particular, using condensed ^87^Rb atoms in the magnetic sublevel *m*_F_ = 2, we measure a magnetic field curvature inside the ISS payload’s vacuum chamber of $\left\vert B^{\prime\prime} \right\vert=\left(614.1\pm 0.3\right)\,{\rm{nT}}\,{{\rm{mm}}}^{-2}$ when analyzing all interferometer times globally. At individual interferometer times 2*T* = 10.3, 12.3, 16.3 ms, we measure magnetic field curvatures with average uncertainties on the order of 5 nT mm^−2^. Deploying *m*_F_ = 0 atoms, we verify the linear Zeeman effect as the leading source of the measured curvature and provide an upper bound to other contributions. Finally, we obtain differential butterfly measurements with *m*_F_ = 2 atoms, consistent with vanishing third-order magnetic field gradients.

We foresee two different applications of the magnetic curvature sensor in space, the first one targeting the in-vacuum characterization of magnetic fields in the aforementioned fundamental physics experiments based on atom interferometry. In such experiments, for instance, when testing general relativity^8–10^, one of the leading systematic effects coupling into the signal are magnetic field gradients. Fully avoiding such couplings is not possible, neither by operating on a magnetically insensitive sublevel due to the non-linear Zeeman effect, nor by advanced compensation techniques^39^. Thus, magnetic field gradients need to be mapped microscopically at the measurement location, i.e., inside the vacuum chamber, where only the atoms have direct access. Devices such as vector fluxgate magnetometers^40^ and scalar atomic magnetometers^41^ generally offer high precision; however, they have to measure from out of vacuum, naturally impeding the performance for gradient reconstruction at the relevant location, especially considering higher-order spatial components. In contrast, utilizing a differential atom interferometric magnetometer enables precise in-vacuum characterization of magnetic field gradients^23–25^ at the right spot. Multiple orders can be mapped out by deploying the differential interferometer at various positions or by adapting the scheme to be sensitive to a different order, as presented in this work. Due to the absence of gravity, performing such characterizations in orbit instead of during an on-ground commissioning phase provides the advantage of more localized measurements under the relevant environmental conditions.

As a second application, deployed on a dedicated satellite platform, we envision the magnetic curvature sensor as part of a multi-modal quantum sensor^23,42^ for various low-frequency geophysical parameters. Here, traditional space magnetometry missions^43–47^ use hybrid systems of vector fluxgates for performing the core measurements and scalar atomic magnetometers as absolute references for calibrating the drifting fluxgates. The number and physical placement of these sensors determine the measurable quantities; e.g., first-order gradients are accessible by placing two fluxgates at a fixed distance. Alternatively, atom interferometry-based magnetometers provide the flexibility to measure different orders with the same hardware, while being inherently drift-free and thus particularly suited for low-frequency signals. The key advantage of deploying atom interferometers in a space mission, however, is their simultaneous susceptibility to gravitational and inertial signals. For instance, while this work demonstrates measurements of second-order magnetic field gradients, the same scheme can be applied to retrieve a first-order gravity gradient^20,21^. Future scenarios in which the multi-modality of atom interferometers is preferred over single-use traditional magnetometers might include experiments for understanding our Earth’s composition^45,48^, interplanetary missions^47,49^ or climate science^50^.

## Results

**Fig. 1:**
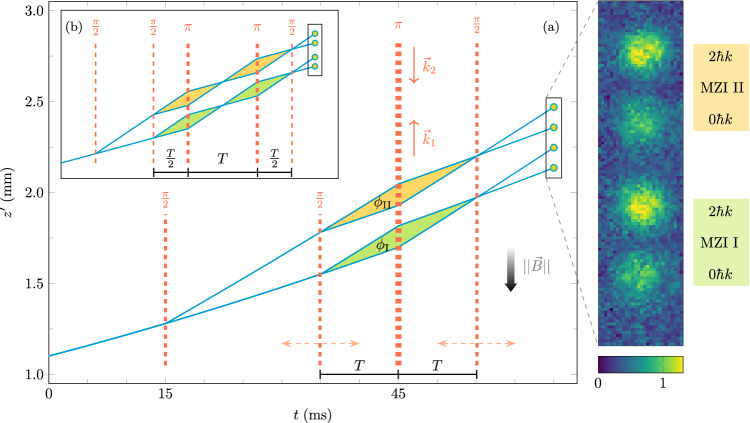
Atom interferometry sequence for measuring differential accelerations. Atom trajectories of ^87^Rb atoms in the *m*_F_ = 2 hyperfine state (blue lines) showing the position during **a** differential Mach–Zehnder-type atom interferometers and **b** differential butterfly interferometers. The interferometers consist of four or five laser pulses (red dashed lines), respectively, with effective wave vector *k*_eff_ = ∣***k***_1_∣ + ∣***k***_2_∣ = 2*k* that coherently manipulate the atoms. Positions are measured in the direction ${z}^{{\prime} }$ of the Bragg beam. **a** A first *π*/2 splitting pulse is applied 15 ms after release from the trap, generating a superposition of atoms in the two momentum states 0*ℏk* and 2*ℏk,* which each serve as input states for a three-pulse Mach–Zehnder interferometer (*π*/2 – *π* – *π*/2-pulse) that again splits, reflects, and recombines the atoms by common laser pulses. After the last laser pulse, the four resulting atom clouds spatially separate during 10–15 ms of free evolution and are detected by a single absorption image (see exemplary density plot for 2*T* = 20.3 ms, size 117 × 488 μm^2^). The total interferometer time 2*T* is varied while the *π*-pulse is always applied 30 ms after the initial splitting pulse. Accelerations of the atoms with respect to the laser beams, caused for instance by spatially dependent magnetic fields ${\boldsymbol{B}}({z}^{{\prime} })$, lead to non-vanishing interferometer phases *ϕ*_I_ and *ϕ*_II_ affecting the relative population in the output ports and enable measurement of differential accelerations between the two MZIs. **b** By adding a second *π* mirror pulse during the interferometer sequence, a differential figure-eight or butterfly geometry can be realized, which in leading order is insensitive to acceleration gradients, but instead reveals acceleration curvatures if present.

We perform two types of differential atom interferometers to characterize the gradient and curvature of the local force field acting on the atoms within CAL aboard the ISS. For characterization of the force gradient, we employ a differential Mach–Zehnder-type geometry defined by the four-pulse sequence shown in Fig. 1a, and apply it separately on atoms in a magnetic-sensitive or magnetic-insensitive state. To characterize the force curvature, we extend this scheme to a double-loop differential butterfly geometry by adding a second mirror pulse as indicated in Fig. 1b. The experiment is based on a cloud of Bose-condensed rubidium atoms that are carefully prepared through a sequence of magnetic traps for free fall at expansion rates below nK. Extracting and comparing the atom numbers from absorption images of individual exit ports of the atom interferometer enables the determination of a differential phase and thus the calculation of various present force gradients and curvatures.

### Experimental setup and atom source preparation

CAL is a space-based BEC machine located aboard the ISS within the US Destiny module and is mounted inside an ISS EXPRESS rack, only a few meters away from the ISS center of mass. Vibration isolation on the ISS is not comparable to ground-based laboratories, and as a consequence, vibrations with acceleration amplitudes up to the order of $1{0}^{-3}\,\mathrm{g}/\sqrt{\mathrm{Hz}\,}$ are present over a wide range of frequencies^36^. The nominal rotation of the ISS at one revolution per orbit ensures that the interferometer beam of CAL always points nadir towards Earth. This rotation is slow enough to not compromise the experiments reported here, and the Sagnac phase caused by the precessing motion of the ISS around the Bragg beam axis is suppressed due to the geometry. As such, CAL constitutes a unique platform for BEC interferometry in space, which has been described in detail in references^32,36,37^.

**Fig. 2:**
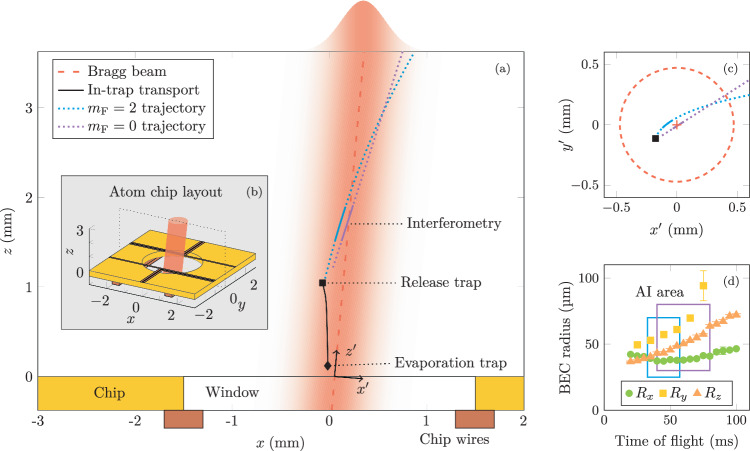
Interferometry setup and atom source preparation of the Cold Atom Lab. **a** Orientation of the Gaussian-shaped Bragg beam (red area) in the *x*-*z*-plane with respect to the atom chip (yellow rectangles) and chip wires (brown rectangles) viewed in the direction of the main imaging axis (*y*-direction). The Bragg beam points towards Earth and enters the vacuum chamber through a window in the atom chip and is retro-reflected by a mirror (not shown). BEC evaporation is performed close to the atom chip in a tight magnetic trap (black diamond), followed by a transport to the shallower release trap (black square) centered above the chip window. Atoms in the magnetic-sensitive *m*_F_ = 2 hyperfine state are released with a non-vanishing velocity away from the atom chip and follow a parabolic trajectory (blue dotted line) caused by magnetic forces. Transferring atoms in the magnetic-insensitive *m*_F_ = 0 hyperfine state by an adiabatic rapid passage 15 ms after release yields an unperturbed linear center-of-mass motion (purple dotted line). Interferometry is performed in a region where the atoms (solid blue and purple lines) are close to the maximum intensity of the Bragg beam. Here, the trajectories during the longest achievable interferometer times 2*T* = 24.3 ms and 2*T* = 40.3 ms, for *m*_F_ = 2 and *m*_F_ = 0 atoms are shown, respectively. **b** 3D layout of the atom chip and the Bragg beam with the chip window in the center. The dotted rectangle shows the boundaries of subplot (**a**). **c** Trajectories of the *m*_F_ = 2 (blue dotted line) and *m*_F_ = 0 (purple dotted line) atoms in the $x^{\prime}$-$y^{\prime}$-plane perpendicular to the Bragg beam ($z^{\prime}$-axis) showing motion of the atoms through the Bragg beam illustrated by its center (red cross) and beam waist (red dashed line). **d** Thomas–Fermi radii of the *m*_F_ = 2 BEC during time of flight after release from the trap. Error bars show the standard deviation of repeated measurements. Blue and purple rectangles indicate when the atom interferometer is applied for atoms in the *m*_F_ = 2 and *m*_F_ = 0 states, respectively.

The CAL atom chip produces a magnetically trapped BEC of ^87^Rb atoms in the *F* = 2, *m*_F_ = 2 hyperfine state with approximately 9 × 10^3^ condensed atoms and a BEC fraction of roughly 70% through evaporative cooling^32,36,51^. For atom interferometry, the atoms must be positioned within the Bragg interferometry beam well above the atom chip as illustrated in Fig. 2. Thus, after evaporative cooling, we magnetically transport the atoms^34,38,52^ within 200 ms over roughly 1 mm in both the *y*- and *z*-directions. This final trap is aligned with the Bragg beam and provides small trap frequencies (*ω*_*x*_, *ω*_*y*_, *ω*_*z*_) = 2*π* × (9.28, 21.2, 18.1) Hz, enabled by the absence of gravitational sag in microgravity.

The CAL interferometry beam has a wavelength of 785 nm and features two adjustable and slightly detuned frequency components, building up two standing and two moving optical lattices. These can be used to couple different momentum states of the ^87^Rb atoms via Bragg transitions^53^. To ensure resonance with only one of the moving lattices, the atoms need a non-zero momentum *p*_0_ along the beam^54^. The beam points in $z^{\prime}$-direction closely aligned with the *z*-axis and tilted by (4.77 ± 0.04)^∘^ towards the *x*-axis, with a beam width of (0.47 ± 0.02) mm, as confirmed by careful calibration measurements of the Bragg beam orientation and intensity (see “Methods”). Any transversal velocity of the atoms relative to the beam should be minimized. We control the atoms’ momentum by modifying the trapping potential during a final hold time of 10 ms and by briefly applying additional magnetic field gradients along the *x*-axis after release. In the *m*_F_ = 2 case, this procedure launches the atoms away from the chip with an initial velocity of (*v*_*x*_, *v*_*y*_, *v*_*z*_) = (1.8, 2.7, 12.5) mm s^−1^ and uncertainties of up to 0.2 mm s^−1^. After this initial procedure, we switch off all chip and coil currents to observe the free propagation of the atom clouds (see Fig. 2d) and obtain a BEC expansion energy *k*_B_*T*_eff_/2 corresponding to an effective temperature *T*_eff_ of only 0.66 nK along the *z*-direction, where *k*_B_ is the Boltzmann constant. Reaching such small expansion energies is essential to avoid inefficiencies in the Bragg transitions due to Doppler shifts. This performance is largely enabled by microgravity, allowing the controlled release from such shallow traps and thus avoiding the need for additional collimation techniques.

While the evaporation and transport happen with magnetically sensitive atoms in *m*_F_ = 2, we perform some atom interferometry experiments in the magnetically insensitive state *m*_F_ = 0. By applying a radio-frequency adiabatic rapid passage^36^ within the first 15 ms after release, we can transfer the atoms into the *m*_F_ = 0 state with high efficiency. Applying the same adjustable release scheme as in the *m*_F_ = 2 case, we realize an initial velocity of the *m*_F_ = 0 atoms of (*v*_*x*_, *v*_*y*_, *v*_*z*_) = (3.0, 1.4, 9.9) mm s^−1^ with similar expansion energy as in the *m*_F_ = 2 case. The trajectories for both hyperfine states are shown in Fig. 2, clearly indicating that the magnetically sensitive state *m*_F_ = 2 is bent by external forces while the *m*_F_ = 0 atoms follow a straight line. In addition, both trajectories are well aligned with the Bragg beam and thus ensure a long spatial overlap between the free-falling atoms and the center of the Bragg beam, leading to improved pulse efficiencies for extended interferometer times. This improvement over previous experimental campaigns is crucial for the success of the following differential interferometers.

### Differential atom interferometry

To generate the differential atom interferometer geometries shown in Fig. 1, we create two spatially separated Mach–Zehnder interferometers (MZIs) and apply all beam splitter and mirror pulses simultaneously on both atomic clouds. We establish their spatial separation by performing a *π*/2 Bragg transition on the atoms with initial momentum *p*_0_ to create an equal superposition of momentum states *p*_0_ (0*ℏ*k) and *p*_0_ + *ℏk*_eff_ (2*ℏ*k). Here, the effective wave vector *k*_eff_ is given by the Bragg beam’s resonant moving lattice, where *k*_eff_ = ∣***k***_1_ − ***k***_2_∣ while ∣***k***_*i*_∣ = 2*π*/*λ*_*i*_ describes the wave vector of two slightly detuned frequency components. The pulse duration is 0.13 ms. During some time of flight, the two momentum states 0*ℏ*k and 2*ℏ*k spatially separate and serve as the input states for MZI-I and MZI-II, respectively. We have analyzed the classical differential center-of-mass (COM) motion between these two momentum states for expansion times up to 150 ms and extracted a non-interferometric result for the acceleration gradient in $z{^\prime}$-direction of ∣Γ∣ = (36.76 ± 1.07) s^−2^ experienced by the *m*_F_ = 2 atoms in the system (see “Methods” and Discussion). This classical measurement also shows a vanishing acceleration curvature.

Each of the two MZIs illustrated in Fig. 1a accumulates, to leading order, a phase *ϕ*_*j*_ = *k*_eff_*a*_*j*_*T*^2^ with *j* = I, II referring to the individual MZIs, the common pulse separation time *T* measured from the center of the pulses, and the local acceleration *a*_*j*_ acting on the atoms along the direction of the Bragg beam. Measuring these phases individually suffers from laser phase and vibrational noise and becomes infeasible for extended pulse separation times *T* in noisy environments, such as CAL^36^. However, by directly extracting the phase difference Δ*ϕ* = *ϕ*_II_ − *ϕ*_I_, common phase-noise contributions cancel out^55,56^ and the evaluation of the differential MZI becomes independent of this constraint. Consequently, from the differential MZI phase Δ*ϕ*, the local acceleration gradient $\Gamma={\boldsymbol{e}}_{z^{\prime}} \cdot {\Gamma}$ which is assumed to be constant over the experiment region can be evaluated according to the analytic phase relation ${\rm\Delta }\phi={k}_{\mathrm{eff}}\Gamma {\rm\Delta }z^{\prime} {T}^{2}$, where $\Delta z{^\prime}$ is the distance between the individual MZIs. In our case, $\Delta z^{\prime}=2{v}_{{\rm{rec}}}{t}_{{\rm{sep}}}$ with the recoil velocity *v*_rec_ = 5.85 mm s^−1^ and the separation time ${t}_{{\rm{sep}}}=\left(30\,{\rm{ms}}-T\right)$ between the two momentum states prior to the three-pulse MZI sequence. This spatial preparation of the respective MZI input states leads to a measurement of the acceleration gradient aligned with the Bragg beam direction ${{\boldsymbol{e}}}_{z{^\prime} }$. While this often is the quantity of interest, by means of other preparation schemes^57^ other gradients ${\boldsymbol{e}}_{j}\cdot {\Gamma}$ could be accessed; however, access to the local acceleration *a*_*j*_ is fundamentally limited to the component along the Bragg beam.

The measurement of Δ*ϕ* is based on determining the respective atom numbers in all four spatially resolved exit ports from a single experimental shot, an example shown in Fig. 1. Comparing the relative populations *N*_rel,*j*_ = *N*_0*ℏk*,*j*_/(*N*_0*ℏk*,*j*_ + *N*_2*ℏk*,*j*_) of the 0*ℏ*k states in both MZIs for a large set of experimental shots yields correlation plots with varying elliptical shape depending on the differential phase Δ*ϕ*. Differential phase values close to 0 or *π* lead to correlation data forming a strongly elongated ellipse with a diagonal as the limiting case, illustrating that both MZIs are fully in-phase or anti-phase, respectively. In contrast, a differential phase Δ*ϕ* = *π*/2 results in circular correlation data. This measurement scheme is independent of phase contributions that act simultaneously on both MZIs since any common phase only shifts the correlation data point around the ellipse, but does not change the form of the ellipse itself. Hence, even if the individual phase scan for a single MZI is washed out due to vibrational phase noise^36^, the differential phase between two such MZIs can be read out reliably from the correlation plot through ellipse fitting^21,58–60^. Moreover, the visibility of the interferometer manifests itself in the maximum vertical and horizontal extent of the fitted ellipse. Integrating the correlation data over one of the two populations gives rise to the typical double-peak distribution used in single MZIs to extract visibilities in the case of washed-out phase scans.

**Fig. 3:**
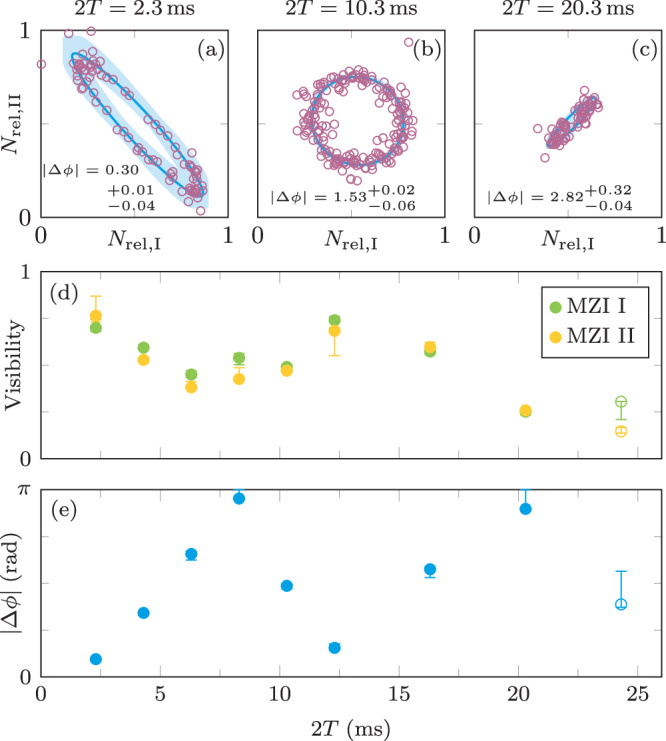
Experimental results of differential Mach–Zehnder atom interferometry in space. **a**–**c** Experimental data of the relative populations *N*_rel,I_ and *N*_rel,II_ in the 0*ℏk* state of both Mach–Zehnder interferometers MZI I and MZI II described in Fig. 1a with atoms in the magnetically sensitive *m*_F_ = 2 state. For growing interferometer time 2*T,* the shape of the correlation data (purple circles) varies between elongated ellipses and circles, indicating a changing differential phase Δ*ϕ* = *ϕ*_II_ − *ϕ*_I_ obtained by ellipse fitting (blue lines with confidence areas in shaded blue) of the data (see “Methods”). **d** Visibility of each MZI is determined by the maximum extend of the ellipse fitted to the correlation data, with error bars showing the 1*σ* ellipse-fitting confidence bounds. Altering visibility is due to day-to-day performance of the experimental apparatus with respect to atom numbers and BEC fraction, and in general decreases for long interferometer times due to motion and expansion of the atoms perpendicular to the Bragg beam (see Fig. 2). **e** Measured differential phase for each interferometer time 2*T* illustrating the presence of differential accelerations. Error bars are based on 1*σ*-confidence bounds of the bootstrap analysis. For 2*T* = 8.3 ms and 2*T* = 20.3 ms the error bars were extended to include *π* since in these cases the data did not allow a clear differentiation from this value (see “Methods”). The data at 2*T* = 24.3 ms is not considered for further analysis due to the poor visibility of less than 15% in MZI II and the resulting large phase uncertainty.

We perform a differential MZI measurement campaign with *m*_F_ = 2 atoms. To this end, we employ nine different interferometer times 2*T*, ranging from 2.3 to 24.3 ms with an average number of 96 experimental shots per interferometer time. Distributed over these shots, we scan the laser phase of the last Bragg pulse from 0 to 2*π* in steps of *π*/6 in order to realize different populations in the respective exit ports. Due to laser phase noise, this discrete manual phase scan becomes blurred and, in principle, even unnecessary for most of the used interferometer times. During the interferometer sequence, the atoms experience an acceleration along the beam direction. To ensure resonance with the mean velocity of the atom clouds, we adjust the Bragg pulse two-photon detunings for each interferometer time and each pulse, individually. The three pulse durations are constant over all sequences at (0.1, 0.2, and 0.1) ms, respectively. In Fig. 3a–c, we exemplary show correlation plots as the measurement outcome for three different interferometer times from this campaign.

We determine Δ*ϕ* and the visibility as well as their uncertainties by fitting a rotated and shifted ellipse to the correlation data of each individual interferometer time (refer to “Methods” for details on the ellipse fitting procedure). The main limitation for extracting the differential phase for long interferometer times stems from the vanishing visibility of population oscillations in the individual MZIs, shown in Fig. 3d. This is caused by the atom’s transverse COM motion relative to the Bragg beam such that the atom cloud explores regions of varying Bragg beam intensity. This effect leads to non-ideal transfer efficiency for long interferometer times and therefore poses a limit for the maximal time 2*T* that could be performed in the *m*_F_ = 2 case.

**Fig. 4:**
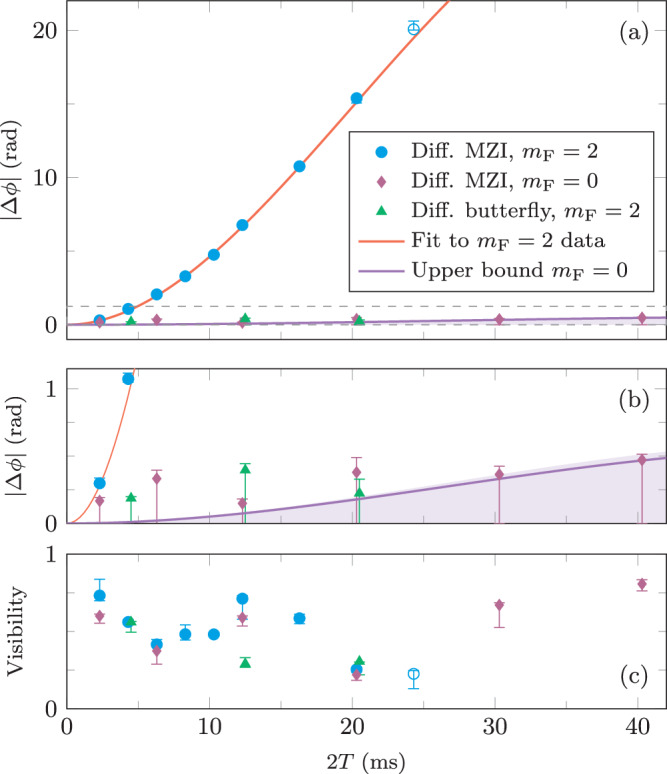
Comparison of complementary differential interferometers. **a** Measured differential phase Δ*ϕ* as a function of the interferometer time 2*T* for the differential MZI sequence shown in Fig. 1a performed with atoms either in the *m*_F_ = 2 (blue dots) or *m*_F_ = 0 (purple diamonds) hyperfine state as well as results for *m*_F_ = 2 atoms in a differential butterfly geometry (green triangles), featuring two *π*-pulses instead of one (see Fig. 1b). Error bars are obtained by ellipse fitting uncertainty (see “Methods”). None of the data sets for the *m*_F_ = 0 and the butterfly interferometer allow a clear differentiation from Δ*ϕ* = 0, such that for these campaigns, the error bars were extended to include zero. **b** Magnification of (**a**) for small values of Δ*ϕ,* illustrating that for *m*_F_ = 0 atoms and for the butterfly interferometer, all measured differential phases are quite small and do not show a clear increasing behavior. A phase relation fit (red) to the *m*_F_ = 2 data (blue) reveals an acceleration gradient ∣Γ∣ = (39.46 ± 0.02) s^−2^. In contrast, the *m*_F_ = 0 data (purple) shows an upper bound for a non-magnetic acceleration gradient based on the longest interferometer time 2*T* = 40.3 ms of $| \Gamma | \le 0.3{1}_{-0.31}^{+0.03}\,{{\rm{s}}}^{-2}$. The butterfly results are compatible with zero-force curvature. Overall, the data show clear evidence of a differential magnetic force acting on the atoms. **c** Visibility of the differential interferometer based on the average of the two corresponding single interferometers. Error bars correspond to the largest individual uncertainty. Visibility of *m*_F_ = 2 measurements decreases for long interferometer times due to magnetic forces moving the atoms through the Bragg beam.

The results of the differential phase fits are displayed in Fig. 3e for all interferometer times 2*T*. Due to multiple ambiguities in inferring the differential phase from ellipse fits, all measurements are limited to absolute phases in between 0 and *π*. This ambiguity prevents the direct determination of the acceleration gradient from individual interferometer times alone. However, by relating the differential phases from all interferometer times, we can evaluate the acceleration gradient. For small interferometer times, 2*T* ≤ 6.3 ms, the measured differential phase values slowly increase and can be connected by the analytic phase relation for Δ*ϕ* to obtain a short-time estimate of Γ. By extending this relation to larger times, we resolve the phase ambiguity for all measurements with 2*T* ≥ 8.3 ms by choosing the best match step by step. Figure 4a shows the resulting differential phases after resolving all ambiguities. The acceleration gradient from this global fit over all interferometer times is then given by ∣Γ∣ = (39.46 ± 0.02) s^−2^.

We also evaluate the force gradient on magnetically insensitive *m*_F_ = 0 atoms. In contrast to the magnetically sensitive state, the *m*_F_ = 0 atoms are not significantly accelerated by residual magnetic field gradients during the interferometer sequence, reducing their transversal motion relative to the Bragg beam as shown in Fig. 2c. This improves the Bragg transition efficiencies for long times of flight, leading to better visibilities for long interferometer times as displayed in Fig. 4c. The approach allows to extend the interferometer time up to 2*T* = 40.3 ms while still observing a signal at the exit ports, ultimately limited by detection noise due to small atom numbers. The measured differential phases are shown in Fig. 4 with no clear dependence on the interferometer time. Based on the fact that all phase values lie well in the small subregion [0, *π*/6], we rule out that a relation to the interferometer time is merely hidden in the ellipse fit phase ambiguity *ϕ*_meas_ ∈ [0, *π*]. Moreover, for all *m*_F_ = 0 campaigns, the correlation data does not allow a clear distinction whether the differential phases are truly non-zero or not and, consequently, the error bars include Δ*ϕ* = 0. Thus, for the *m*_F_ = 0 atoms, we evaluate an upper bound of the acceleration gradient based on only the longest interferometer time 2*T* = 40.3 ms, yielding a value of $| \Gamma | \le 0.3{1}_{-0.31}^{+0.03}\,{{\rm{s}}}^{-2}$.

We further extend the differential MZI configuration to a differential butterfly interferometer (BFI) to measure force curvatures instead of gradients. The differential BFI consists of two spatially separated butterfly configurations^61^ as shown in Fig. 1b. Compared to the differential MZI, it features an additional mirror pulse before the closing beam splitter. The resulting symmetry eliminates the leading term that dominated the differential MZI signal, such that it reveals smaller contributions due to force curvatures (see “Methods”). We perform a measurement campaign with the differential BFI using *m*_F_ = 2 atoms. For three different interferometer times 2*T* ranging from 4.5 to 20.5 ms, we again see no clear relation between the differential phase and the interferometer time, as shown in Fig. 4. Noise in the relative port population makes an extraction of Δ*ϕ* close to degeneracy of the ellipse challenging, but the obtained results are compatible with zero force curvature Γ_I_ − Γ_II_ ≈ 0 which agrees well with our classical COM measurement (see “Methods”). Although anharmonic potentials also affect the differential MZI signal, our butterfly measurements indicate that accounting solely for the leading-order terms above, that is, the magnetic field curvature, is sufficient for analyzing the differential MZIs.

### Magnetometry

Combining the results from all measurement campaigns, we locally characterized the magnetic field. The acceleration gradient observed in the differential MZI with magnetically sensitive *m*_F_ = 2 atoms almost fully vanishes for magnetically insensitive *m*_F_ = 0 atoms, highlighting its magnetic nature. We thus conclude the presence of a local magnetic field curvature along $z^{\prime}$ of $\left\vert B^{\prime\prime} \right\vert=\left\vert \Gamma \cdot {m}_{{\rm{Rb}}}/\left({m}_{F}{g}_{F}{\mu }_{B}\right)\right\vert=(614.1\pm 0.3)\,{\rm{nT}}\,{{\rm{mm}}}^{-2}$ with less than 0.8% of contributions outside of the linear Zeeman effect quantified through the upper bound of Γ acting on *m*_F_ = 0 atoms. Furthermore, our differential BFI measurement is consistent with the third spatial derivative of the magnetic field being zero.

Extending our campaign to quantify other orders of the magnetic field is partially possible by adapting the interferometer scheme. A non-differential scheme would directly access the gradient of the absolute magnetic field in the beam direction ${\partial }_{z{\prime} }\left\vert B\right\vert$. However, in the current experimental setup^36^ laser phase and vibrational noise would limit the interrogation time and thus, the achievable sensitivities prohibit meaningful analyses. This limitation can be overcome in future experiments with vibration isolation schemes^62^. Accessing the absolute value of the field directly is not trivially possible due to the inherent nature of atom interferometers to measure differences in the potential rather than magnetic fields directly. Extensions towards such quantities rely on differential interferometers between different magnetic states^23^.

**Fig. 5:**
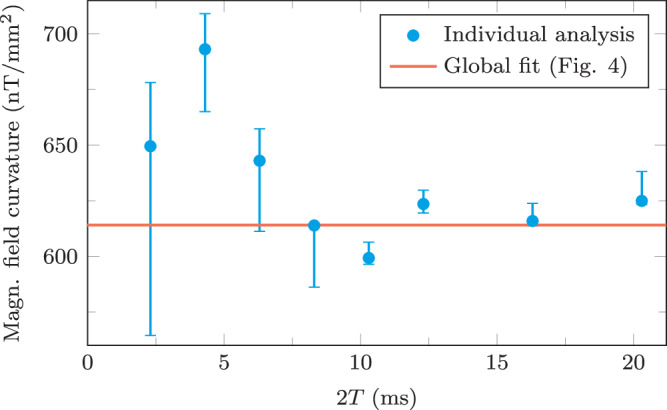
Measured magnetic field curvature. Using the data from the differential MZI measurement performed on *m*_F_ = 2 atoms, for each interferometer time 2*T*, we individually infer the magnetic field curvature $\left\vert B^{\prime\prime} \right\vert=\left\vert \Gamma \cdot {m}_{{\rm{Rb}}}/\left({m}_{F}{g}_{F}{\mu }_{B}\right)\right\vert$ (blue dots). Individual error bars are calculated through error propagation. The global fit shown in Fig. 4 gives $\left\vert B^{\prime\prime} \right\vert=\left(614.1\pm 0.3\right)\,{\rm{nT}}\,{{\rm{mm}}}^{-2}$ (red line). The weighted average for the individual measurements is $\left\vert B^{\prime\prime} \right\vert=\left(622\pm 20\right)\,{\rm{nT}}\,{{\rm{mm}}}^{-2}$. The best individual performances are obtained for interferometer times ranging from 2*T* = 10.3 to 16.3 ms, including 65 to 181 shots, respectively, resulting in average uncertainties in the magnetic field curvatures of 5 nT mm^−2^.

Instead of inferring the differential acceleration by fitting Δ*ϕ* as a function of *T* for the entire data set, we can also calculate it directly for each value of *T*, analyzing each interferometer time individually. The global analysis resolved the ambiguity in the differential phases Δ*ϕ*^MZI^ measured with the *m*_F_ = 2 atoms. Using the same phase relation as before, the acceleration gradient Γ and thus the magnetic field curvature *B″* is determined via the linear Zeeman effect. Fig. 5 shows the resulting curvatures for individual interferometer times. The most sensitive measurements are realized for interferometer times ranging from 2*T* = 10.3 to 16.3 ms, featuring magnetic field curvatures with average uncertainties of 5 nT mm^−2^. For example, we measure $\left\vert B^{\prime\prime} \right\vert=59{9}_{-3}^{+7}\ \,{\rm{nT}}\,{{\rm{mm}}}^{-2}$ using 2*T* = 10.3 ms. This experiment includes 181 shots with an average atom number of 4.5 × 10^3^ atoms per MZI and visibility of 0.48. Based on these numbers, the corresponding 1*σ*-shot-noise limit for the magnetic field curvature amounts to 0.4 nT mm^−2^. The gap between the experimentally achieved sensitivity and the theoretical limit can have a variety of causes. In particular, we suspect signal and experiment drifts in between shots, noisy imaging at small atom numbers, and consequently larger uncertainties from the ellipse-fitting process.

In Fig. 5, we observe a spread of the individually analyzed data around the globally fitted result and between each other. This results in a weighted average for the individual interferometer times of $\left\vert B^{\prime\prime} \right\vert=\left(622\pm 20\right)\,{\rm{nT}}\,{{\rm{mm}}}^{-2}$ with its standard deviation being larger than most individual uncertainties. To understand this spread, we have performed a detailed numerical modeling taking into account more experimental parameters, such as finite pulse durations^63^ as well as additional phase contributions within a quadratic potential^64–67^. Comparing these numerical results to the ones from our analytical phase relation reveals only a small offset on the order of a few nT mm^−2^ while not decreasing the observed spread, confirming the validity of our simpler analytical phase model. Furthermore, higher-order contributions beyond a quadratic potential were constrained by our differential BFI measurements already, and including these in our phase model does not yield a consistent and improved explanation of the data (see “Methods”). As the experiments were performed over a duration of several months, we conclude that actual drifts in the magnetic field curvature are the most likely explanation for the observed data spread. While with CAL as a multi-user facility, such long campaign durations are hard to avoid, this does not constitute a general limitation of the methods presented here when deployed in dedicated magnetometry missions.

## Discussion

In this article, we have performed various differential atom interferometer sequences based on Mach–Zehnder and butterfly geometries to characterize the magnetic field inside the CAL vacuum chamber onboard the ISS. In particular, we have measured the residual magnetic field curvature with a 1*σ*-sensitivity of 0.3 nT mm^−2^ and have obtained complementary measurements with magnetically insensitive atoms to confirm the magnetic nature of the measured force gradients. Furthermore, we have verified through the differential butterfly interferometer that there is no significant contribution from a third spatial derivative of the magnetic field acting on the atoms, and the measured differential forces can be attributed to a magnetic field curvature. With these results, we have successfully realized a BEC-based magnetometer in space, representing an important milestone for space-based quantum sensors in general.

The residual magnetic fields present within the CAL device have been observed earlier by studying the COM motion of the atoms^34,68^ and can best be explained by magnetic fields originating from the atom-chip connectors or other magnetized parts of the experimental setup inside the magnetic shield. Their precise characterization has only been possible by applying interferometry techniques as presented in this work. The measured magnetic field curvature corresponds to an effective harmonic trap frequency of ${\omega }_{z^{\prime} }=2\pi \times (1.000\pm 0.002)\,{\rm{Hz}}$ along the direction of the Bragg beam and thus, would influence both the atom’s COM motion as well as their expansion dynamics when employed for longer times required for precision sensing campaigns.

Comparing the magnetic field curvature result from our differential interferometer of $\left\vert B^{\prime\prime} \right\vert=(614.1\pm 0.3)\,{\rm{nT}}\,{{\rm{mm}}}^{-2}$ to the result obtained from the difference of the classical COM motion of atoms in the 0*ℏ*k and 2*ℏ*k state of $\left\vert B^{\prime\prime} \right\vert=(572\pm 17)\,{\rm{nT}}\,{{\rm{mm}}}^{-2}$ illustrates the higher sensitivity of the interferometer in comparison to a classical measurement. The existing offset between both approaches can be attributed to the fact that the interferometer measures much more locally than the classical measurement. The interferometer takes place only during a small fraction of the total atom cloud trajectory (0.43 mm motion during a 2*T* = 20.3 ms interferometer with a separation of 0.23 mm compared to 3.5 mm during 130 ms differential time-of-flight) and thus introduces less averaging.

Our experiments demonstrate an in-vacuum magnetic field characterization, mandatory for any future space-deployed high-precision gravity measurement^29,30^ or fundamental physics test^8^ to estimate unwanted phase shifts. In most cases, the magnetic field environment is mapped out by Ramsey interferometer techniques that allow for a determination of the magnetic field gradient in a certain spatial region^27,30^. In contrast, our approach also enables a direct measurement of the higher derivatives of the magnetic field and thus provides deeper insights into the local magnetic field environment of the apparatus. Furthermore, our differential interferometer can operate in a much smaller volume compared to Earth-bound measurements and thus improves the spatial resolution of the magnetic field characterization.

In order to achieve the results presented in this article, a detailed characterization of the Bragg beam orientation and intensity, as well as the atom source, has been crucial to enable the required spatial overlap between the atom cloud and the Bragg beam during the interferometer. In this way, we have substantially improved the single-mirror pulse efficiencies up to 85%, outperforming previous atom interferometer implementations of CAL^36,37^. This Bragg beam characterization constitutes another Bragg-pulse-enabled quantum sensor used for validation of the experimental setup by itself.

In addition, the microgravity conditions aboard the ISS have enabled our measurements in the following ways: First, a freely falling laboratory allows to probe the atoms for long times at a constant position or close to a specific object, facilitating characterization of the system and sensing applications. In our case, the atoms moved by only 0.43 mm during each MZI for an interferometer time of 2*T* = 20.3 ms and have thus enabled a very localized determination of the magnetic field curvature. Performing the same measurement on Earth would result in a total motion of 9.0 mm between the beam-splitting pulses of the MZI, such that the measurement signal would be averaged over a longer distance, reducing the spatial resolution. Second, microgravity allows to utilize shallow release traps to reduce the expansion energy of the BEC and to enable compact atom clouds even after long free evolution times. By carefully designing the transport of the BEC to the release trap and optimizing the release scheme, we have achieved BEC expansion energies of 0.66 nK, which are much lower compared with the proof-of-principle interferometry demonstrations on CAL. Consequently, we have been able to increase the pulse efficiency of the Bragg transitions and thus extend the interferometer times to 2*T* = 40.3 ms. The achieved expansion rates are sufficiently low to not constitute the limiting factor during our campaign, rendering the application of delta-kick collimation techniques (DKC)^33,34,69–71^ unnecessary. However, if the atom’s COM motion transversal to the beam would be eradicated as a limiting factor, smaller expansion velocities along the beam would enable improved Bragg transfer efficiencies, while smaller transversal expansion velocities would lead to more homogeneous excitation during pulses.

To further improve sensitivities and fully exploit microgravity conditions, future space-based experiments^56^ should aim to mitigate the following limitations. One of the leading limitations in our setup has been the small total number of condensed atoms. Routinely having an average of roughly 2 × 10^3^ atoms per exit port renders the atom number determination through absorption imaging challenging, especially when interference reduces the actual atom number well below 10^3^ in single exit ports. These low numbers introduce noise in the correlation data and increase the uncertainty of phase estimation. Increasing the total number of condensed atoms from around 10^4 ^to 10^6^, as for instance, envisioned for the BECCAL apparatus^72^, would improve the signal-to-noise ratio considerably. Compared to the present instrument, this can be achieved by hardware upgrades to the atom chip assembly targeted at more efficient magnetic trap loading and evaporation. Furthermore, the Bragg beam properties and alignment with the atom trajectories could be improved to preserve the contrast of the entire interferometer for longer times. In particular, a larger Bragg beam diameter would minimize the effects of the whole atom cloud moving transversal to the beam due to magnetic forces and reduce losses during the pulses. Similarly, a higher laser power would enable much shorter beam splitter and mirror pulses, increasing their efficiencies. Lastly, the Bragg beam experiences diffraction effects from the edge of the chip window and the chip wires, compromising the uniformity of the beam. Adjusting the beam path could reduce these unwanted effects and further improve the pulse efficiencies. These aspects are the main reasons why our implementation did not reach the sensitivities of dedicated ground-based differential atom interferometry setups^21,73^, that are less limited by space, weight, and power constraints. Although not limiting for our interferometry configuration, for non-differential schemes a vibration isolation to reduce laser phase noise and compensate residual signals coupling in through the retro-reflection mirror^62^, as well as a tip-tilt mirror to compensate platform rotations at significantly longer times, are imperative.

Such improved atom interferometer setups would enable the measurement of geophysical parameters competitive with traditional space missions. State-of-the-art drift-stabilized fluxgate sensors for measuring magnetic field vectors from a satellite platform^45,46^ perform at a sensitivity of $11\; \text{ to }\; 25\,{\rm{pT}}/\sqrt{{\rm{Hz}}}$ @ 1 Hz. However, solely measuring the absolute field via scalar atomic magnetometers enables sensitivities down to $1\,{\rm{pT}}/\sqrt{{\rm{Hz}}}$ over a broad range of frequencies^45^. Positioning multiple scalar magnetometers, analogous to the usual positioning of multiple fluxgates^46^ at a distance of 40 cm, would enable the measurement of a second order magnetic field gradient with a sensitivity of $15\,{\rm{pT}}/{{\rm{m}}}^{2}/\sqrt{{\rm{Hz}}}$. Analogously, a differential atom interferometer with performance parameters envisioned for future fundamental physics tests^9^, using an equivalent separation of 40 cm, would be shot noise limited at $0.06\,{\rm{pT}}/{{\rm{m}}}^{2}/\sqrt{{\rm{Hz}}}$ @ ≲ 0.1 Hz, while simultaneously being sensitive to gravity gradients at 5 mE @ ≲ 0.1 Hz. Measuring signals at larger frequencies with more conservative parameters of 10^5^ atoms per interferometer and a separation time of *T* = 250 ms could be possible at a sensitivity of $180\,{\rm{pT}}/{{\rm{m}}}^{2}/\sqrt{{\rm{Hz}}}$ @ 1 Hz which is compelling for a space-deployed multi-modal quantum sensor already in the intermediate term.

To conclude, our magnetometer campaign aboard the ISS constitutes the measurement of a residual field based on BEC interferometers in space. Thus, this campaign represents a major advance for space-based quantum sensing and paves the way for the next generation of multi-user BEC facilities in orbit^72^ as well as serving as a true pathfinder for more sophisticated missions in the future, like searching for gravitational waves or performing Earth observation from space^3,4^.

## Methods

### Characterization of Bragg transitions and beam orientation

We manipulate the atom’s momentum states with a Bragg laser beam at 785 nm operated with box-shaped pulses. The maximum laser output power of 66 mW is split into two frequency components with adjustable wave vectors ***k***_1_ and ***k***_2_ where ∣***k***_*i*_∣ = 2*π*/*λ*_*i*_. By controlling the two-photon detuning Δ between the two laser frequency components, we enable resonant Bragg transitions only between an initial momentum *p*_0_ (0*ℏ*k state) and the momentum *p*_0_ + 2*ℏ*k (2*ℏ*k state). Here we take into account both the recoil effect on the atoms and the Doppler shift to identify the resonant detuning, such that transitions to other momentum states are well suppressed^54^.

The transfer efficiency between the two momentum states 0*ℏ*k and 2*ℏ*k strongly depends on the local Bragg beam intensity. It is therefore particularly important to carefully characterize the Bragg beam to optimize the diffraction efficiency at different positions. As CAL is fully remote- controlled and encapsulated during operation, it is not possible to measure the beam orientation or intensity from the outside and to confirm its specifications after installation onboard the ISS. Taking advantage of the adjustable release scheme described in the results section, we realized different trajectories for atoms in the *m*_F_ = 0 and *m*_F_ = 2 states. The trajectory of the atom cloud was determined by recording the center-of-mass position of the atoms for different time of flights using both camera systems. For each trajectory and center-of-mass velocity, we determined the optimal detuning Δ via individual resonance scans, yielding values ranging from 40 kHz to 55 kHz. In each case, we applied a single Bragg pulse with varying duration from 0.1 to 1.6 ms to drive Rabi oscillations, where the lower limit of 0.1 ms was set by technical constraints. By measuring the populations in the 0*ℏ*k and 2*ℏ*k states after the Bragg pulse and taking into account the BEC expansion rate and pulse shape, we obtain the local Rabi frequency of the laser beam at the respective BEC positions during the pulse.

Since the Rabi frequency is proportional to the intensity of the Bragg laser which features approximately a Gaussian intensity profile, the local Rabi frequency depends on the transverse position and we expect the form

1 $$\Omega ({x}^{{\prime} },{y}^{{\prime} })={\Omega }_{\max }\,{{\rm{e}}}^{-2[{({x}^{{\prime} }-{x}_{0}^{{\prime} })}^{2}+{({y}^{{\prime} }-{y}_{0}^{{\prime} })}^{2}]/{w}_{0}^{2}}$$

with the maximum Rabi frequency ${\Omega }_{\max }$ as well as the width *w*_0_, and the centers $x^{{\prime} }_{0}$ and $y^{{\prime} }_{0}$ in $x^{\prime}$- and $y^{\prime}$-direction of the Bragg beam. Since there is a small angle *α* between the Bragg beam and the *z*-axis in the *x*-*z*-plane, the coordinates transform in the following way

2a $${x}^{{\prime} }=x\cdot \cos \alpha+z\cdot \sin \alpha$$

2b $${y}^{{\prime} }=y$$

2c $${z}^{{\prime} }=-x\cdot \sin \alpha+z\cdot \cos \alpha .$$

By fitting the model function, Eq. 1, together with the coordinate transformations, Eq. 2, to the Rabi scan data, we obtain the maximum Rabi frequency ${\Omega }_{\max }=2\pi \cdot (2.72\pm 0.12)\,{\rm{kHz}}$ and the following geometric parameters: the beam width *w*_0_ = (0.47 ± 0.02) mm, the beam center *x*_0_ = (48 ± 135) μm and *y*_0_ = (92 ± 33) μm at *z* = 0 as well as the angle in the *x*-*z*-plane *α* = (4.08 ± 3.87)^∘^. Here, the relatively large error for *α* is due to the fact that most measurements were performed at similar *z*-positions to better sample the transverse shape of the Bragg beam. The intensity profile of the Bragg beam and the positions of the individual Rabi scan measurements are displayed in Supplementary Fig. S1.

We have achieved maximum transfer efficiencies of 85% for single-mirror pulses performed close to the beam center, showing an improvement compared to previous pulse efficiencies demonstrated with CAL^37^. This result is in good agreement with the maximum theoretical mirror pulse efficiency of 86% determined by the measured Bragg beam intensity and the momentum width of the atom cloud. The pulse efficiency could be directly improved by a larger beam power. Additionally, using advanced Bragg pulses designed via quantum optimal control^74^ could be explored in the future; however, the current instrument software prohibits such methods due to limited phase control in time.

A complementary approach to measure the angle of the Bragg beam more precisely relies on the comparison of the COM trajectories of the 0*ℏ*k and 2*ℏ*k states in a single experimental run. The distance of the atom clouds in these two states in the *x*-*z*-plane is shown in Supplementary Fig. S2 for expansion times up to 150 ms, enabling a very sensitive determination of the Bragg beam angle of (4.77 ± 0.04)^∘^. By additionally observing the atoms in the *x*-*y*-plane, we observe no significant momentum transfer by the Bragg beam in the *y*-direction, as expected.

As a last characterization, we employed the differential COM motion of the 0*ℏ*k and 2*ℏ*k states to verify the camera magnification in-flight. The total transferred velocity 2*ℏ*k/*m* = 11.70 mm s^−1^ is well defined and can be compared with the observed differential velocity measured with an uncertainty of only 0.02 mm s^−1^. This measurement suggests a correction of 6.2 ± 0.2% with respect to the original calibration of the camera system. Notably, when comparing the results of a differential interferometer for such changes of the magnification, there is only a negligible influence on the relative atom numbers in the exit ports and the measurement data extracted from them. Hence, our interferometric results are independent of the actual magnification of the camera system.

### Analysis of absorption imaging data

The raw data of the absorption imaging of both detection systems was analyzed in the following way. For the initial calibration of the atom source generation with short times-of-flight a bimodal fitting procedure was used to consider both the BEC and the thermal clouds. For this purpose, a two-dimensional (2D) Thomas-Fermi and a 2D-Gaussian density profile were superimposed and fitted to the absorption data together. In this way, the atom numbers, positions, and sizes of the clouds were extracted, and information on the BEC fraction was obtained.

For longer free evolution times, and in particular for the interferometry measurements, the thermal cloud was hard to distinguish from the background noise. Thus, only a 2D-Thomas-Fermi profile was used in these cases to obtain atom numbers, central positions, and Thomas-Fermi radii.

Furthermore, in the case of the differential interferometers used to measure the magnetic field curvature, the whole data set was post-processed by a principal component analysis^75^ in order to remove background imaging noise and obtain a better signal-to-noise ratio.

### Classical potential curvature measurement

By performing only a single beam splitting pulse at *t* = *t*_0_ one can observe the trajectories of atoms in the 0*ℏ*k and 2*ℏ*k states simultaneously for long times. The differential motion of these two clouds reveals information about the differential velocity Δ*v* = *v*_2*ℏ*k_ − *v*_0*ℏ*k_ as well as the potential curvature Γ acting differentially on both clouds, i.e., the trapping frequency $\omega=\sqrt{\Gamma }$ of any residual harmonic potential. Starting from the classical equations of motion in a harmonic potential and by taking into account that both clouds are at the same position at *t*_0_ we obtain the distance

3 $$\Delta {x}_{j}=\frac{\Delta {v}_{j}}{{\omega }_{j}}\sin \left({\omega }_{j}(t-{t}_{0})\right)\qquad \,\mathrm{for}\,\quad t > {t}_{0}$$

between both clouds in any spatial dimension *x*_*j*_ = *x*, *y*, *z* and associated velocities *v*_*j*_ and frequencies *ω*_*j*_.

The differential motion can be used to measure the residual trapping frequencies *ω*_*j*_. The total distance between the two split *m*_F_ = 2 atom clouds, as well as the distance per spatial direction, are shown in Supplementary Fig. S2. Using Eq. 3, we obtain ${\omega }_{{\rm{COM}},{z}^{{\prime} }}=2\pi \times (0.965\pm 0.014)$ Hz for the harmonic trapping frequency along the direction of the Bragg beam, or a potential curvature Γ = 36.76 ± 1.07 s^−2^. The fit shows an extremely high coefficient of determination of *R*^2^ = 0.9999, such that the dynamics of the atom clouds can be fully explained by the harmonic potential. In particular, there is no indication of higher spatial derivatives of the potential influencing the dynamics.

Complementary measurements with atoms in the *m*_F_ = 0 state reveal no additional forces acting on the atoms, such that the potential curvature measured for the *m*_F_ = 2 atoms can be attributed to a magnetic field curvature in line with the differential interferometric measurements. Thus, the non-interferometric result for the corresponding magnetic field curvature based on the linear Zeeman effect is $\left\vert B^{\prime\prime} \right\vert=(572\pm 17)\,{\rm{nT}}\,{{\rm{mm}}}^{-2}$. In addition, the non-interferometric COM motion also supports the finding of a vanishing third spatial derivative of the magnetic field from the differential butterfly interferometers.

To ensure consistency of the measurement campaign and potential curvature stability over time, we have repeated the differential center-of-mass campaign shown in Supplementary Fig. S2 after approximately 1 year. The results of both campaigns are in excellent agreement with each other.

### Ellipse fitting

For the differential interferometers reported here, we have measured the relative populations *N*_rel,*j*_ = *N*_0*ℏk*,*j*_/(*N*_0*ℏk*,*j*_ + *N*_2*ℏk*,*j*_) of the 0*ℏ*k states in both MZIs labeled by *j* = I, II through absorption imaging for different laser phase values. Since the Bragg pulses act simultaneously on both MZIs the corresponding populations can be described by the relations

4 $${{\mathcal{N}}}_{{\rm{rel,I}}}={{\mathcal{N}}}_{\mathrm{I},0}+\frac{{{\mathcal{V}}}_{{\rm{I}}}}{2}\cos \left({\phi }_{{\rm{I}}}\right),$$

5 $${{\mathcal{N}}}_{{\rm{rel,II}}}={{\mathcal{N}}}_{\mathrm{II},0}+\frac{{{\mathcal{V}}}_{{\rm{II}}}}{2}\cos \left({\phi }_{{\rm{I}}}+\pi+\Delta \phi \right),$$

where ${{\mathcal{N}}}_{j,0}$ is the offset population, ${{\mathcal{V}}}_{j}$ the visibility and Δ*ϕ* = *ϕ*_II_ − *ϕ*_I_ the differential phase between both MZIs. The additional phase of *π* for the second interferometer is due to the fact that the input for this interferometer is an atom cloud in the momentum state 2*ℏ*k rather than 0*ℏ*k.

In a noisy environment, such as the ISS, it is hard to extract the phase of a single interferometer independently, which is why we consider the correlated data sets and fit both model functions, Eqs. 4 and 5, simultaneously to the data. Consequently, there are five fit parameters which for convenience we stack into the parameter vector $\theta={\left({{ \mathcal N }}_{\rm{I},0},{{{ \mathcal V }}}_{{\rm{I}}},{{{ \mathcal N }}}_{\mathrm{II},0},{{{ \mathcal V }}}_{\mathrm{II}},{\Delta }\phi \right)}^{T}$.

In order to obtain the optimal values for these parameters we perform a nonlinear least square optimization based on the loss function

6 $$L(\theta )=\frac{1}{{N}_{\mathrm{data}}}\sum _{k=1}^{{N}_{\mathrm{data}}}\,\mathop{\min }\limits_{l=1,\ldots,{N}_{\mathrm{disc}}}\,{{\rm\Delta }}_{k,l}^{2}+\frac{1}{{N}_{\mathrm{disc}}}\sum _{l=1}^{{N}_{\mathrm{disc}}}\,\mathop{\min }\limits_{k=1,\ldots,{N}_{\mathrm{data}}}\,{{\rm\Delta }}_{k,l}^{2}.$$

Here, *N*_data_ is the number of experimental data points and *N*_disc_ is the number of points used to discretize the model functions ${{\mathcal{N}}}_{{\rm{rel,I}}}$ and ${{\mathcal{N}}}_{{\rm{rel,II}}}$ for equally distributed values *ϕ*_I_ ∈ [0, 2*π*]. The distance between the individual experimental data points and the discretized points of the ellipse is given by the matrix entries Δ_*k*,*l*_. Hence, the loss function, Eq. 6, is determined both by the minimum distance between each experimental data point and the nearest point of the model functions (first sum) as well as the minimum distance between each discretization point and its nearest experimental data point (second sum).

This loss function approach ensures that closely clustered data points are not mistakenly identified as parts of a larger ellipse, particularly when these points cover only a small segment of the fitted ellipse’s total circumference. Furthermore, the method takes into account the uneven spacing of measurement points due to the position-dependent curvature of an ellipse. This consideration prevents regions of lower curvature, where points are less densely packed, from being underweighted, thus avoiding an inaccurately narrow fit for the ellipse. This aspect is especially critical for data that is strongly correlated (Δ*ϕ* ≈ 0) or anti-correlated (Δ*ϕ* ≈ *π*), where there is a risk of the data being erroneously fitted to a very large ellipse representing an effectively straight line in the correlation plot.

Another effect has to be taken into account for strongly correlated (Δ*ϕ* ≈ 0) or anti-correlated (Δ*ϕ* ≈ *π*) data as well. In the ideal case, a vanishing differential phase would lead to data populating a diagonal in the correlation plot. However, in the presence of detection noise, the experimental data is spread around this diagonal, such that any ellipse fit would yield a non-zero Δ*ϕ* value because a slightly opened ellipse fits the data better and minimizes the loss function. Hence, it is inherently hard for ellipse fitting techniques to distinguish between truly non-zero differential phase values and vanishing differential phases^21,58–60,76^. As a consequence, we extend the error bars to include 0 if the differentiation cannot be done reliably. The same holds true for values close to *π*. The data shown in Fig. 3a–c illustrate this point. On the one hand, in Fig. 3a the points form a slightly opened ellipse with no points populating its center, yielding a truly non-zero differential phase. On the other hand, in Fig. 3c, this assessment cannot be done reliably.

In principle, the problems associated with Δ*ϕ* ≈ 0 or Δ*ϕ* ≈ *π* could be solved through a suitable frequency shift of the detuning Δ from the excited state for the *π*-pulse. This method, which was proposed in ref.^66^ to compensate for the effects of gravity gradients and has been successfully demonstrated in gravity gradiometry measurements^77^ as well as tests of the universality of free fall^8,78^, can also be exploited to add an artificial contribution to the differential phase shift, as done in ref.^79^. In our case, however, the separation between the two interferometers is about a hundred times smaller (0.3 mm compared to 3 cm), and the relevant interferometer times are shorter by a factor of 2 or more. The required frequency jump of the detuning Δ for the *π*-pulse is inversely proportional to the separation and the square of the interferometer time, and it is therefore 400 times higher or more in our case, which amounts to a frequency shift of order 10^2^ GHz and is not a viable option with our setup.

The visibilities of both interferometers are obtained through the same ellipse fitting procedure and correspond to the maximum vertical and horizontal extent of the ellipses. The results shown in Fig. 3d reveal decreasing visibilities for growing interferometer times until the small visibilities significantly impede the differential phase determination, reflected in increasing uncertainty of the differential phase. For the 2*T* = 24.3 ms campaign, the visibility of MZI-II drops below 0.15, rendering the phase estimation unreliable, such that we are not considering the outcome of this campaign for further quantitative analysis.

Experimental correlation data for the differential MZI with *m*_F_ = 0 atoms as well as for the differential butterfly geometry with *m*_F_ = 2 atoms are shown in Supplementary Fig. S3. In these cases, all ellipses are rather elongated, and a clear differentiation between zero and non-zero differential phase values could not be done, such that the error bars include zero differential phase in all campaigns.

Due to the non-differentiability of the loss function *L*(*θ*), the Hessian or curvature matrix, which at the optimum parameters θ^*^ is proportional to the inverse of the covariance matrix, cannot be obtained analytically. Numerical approximations of the Hessian matrix at the optimum show limited robustness to slight variations in the values of the optimal parameters, making the precise determination of error and confidence bounds challenging. In order to nevertheless estimate the parameter errors properly, we perform a bootstrap analysis of the correlated experimental data *N*_rel,I_ and *N*_rel,II_. For this purpose, we sample a number of *N*_BS_ datasets by drawing *N*_data_ data points with replacement from the experimental data. Next, we fit the model functions Eqs. 4 and 5 again to each of the *N*_BS_ synthetically generated bootstrap data sets and obtain the optimal parameters in each case. The resulting statistical distribution of the five fit parameters *θ* is then analyzed for measures like the standard deviation or confidence intervals, where the 1*σ*-confidence bounds obtained in this way are used as the experimental uncertainties in determining the differential phase Δ*ϕ*. Exemplary histograms for the bootstrap analysis of differential interferometers with interferometer times 2*T* = 2.3, 10.3, and 20.3 ms are shown in Supplementary Fig. S4.

### Phase determination including potential contributions beyond second order

The treatment presented in the main text assumes instantaneous laser pulses and quadratic magnetic potentials. We have also performed a more general modeling, which we summarize in this section.

On the one hand, we have modeled the effects of finite pulse duration (taking into account the actual Rabi frequencies and two-photon frequency detunings employed for each pulse) by solving the Schrödinger equation that governs the evolution of the atomic wave packets during each pulse and includes the optical potential associated with the laser field. Our treatment extends the results^63^ for finite pulse duration. Furthermore, in order to include the effects of finite pulse durations in treatments for gravity gradients that originally assumed instantaneous pulses, we consider the freely falling frames co-moving with the mid-point trajectory of each interferometer^80^.

On the other hand, we have considered the effects of potential contributions beyond second order. Here, we show the more complete phase terms and later justify our simplifications. Including gradients up to third order, we write the potential along the Bragg beam direction ${z}^{{\prime} }$ as

7 $$V={V}_{0}+m\left(-{a}_{0}\,{z}^{{\prime} }+\frac{{\Gamma }_{0}}{2}\,{z}^{{\prime} 2}-\frac{{\gamma }_{0}}{6}\,{z}^{{\prime} 3}\right)$$

with a potential offset *V*_0_, the mass *m* of a ^87^Rb atom and the uniform acceleration *a*_0_, acceleration gradient Γ_0_ and acceleration curvature *γ*_0_. The phase of a MZI in a quadratic potential has been calculated in a number of references^64–66,81,82^ and the result is given by

8 $${\phi }_{{\rm{MZI}}}/{k}_{{\rm{eff}}}=a{T}^{2}-\left({v}_{0}+{v}_{{\rm{rec}}}\right)\Gamma {T}^{3}-\frac{7}{12}a\Gamma {T}^{4}+{\mathcal{O}}\left({\Gamma }^{2}\right)$$

with the pulse separation time *T*, the single-photon recoil velocity *v*_rec_, and the position (velocity) *z*_0_ (*v*_0_) of the atoms entering the interferometer. We now expand up to second order the full potential including the acceleration curvature *γ* for the two spatially separated MZI-I and MZI-II with initial positions ${z}_{0}^{I},{z}_{0}^{II}$ and initial velocities ${v}_{0}^{I},{v}_{0}^{II}$, respectively. In this description, we choose the initial velocities to describe the lower path for both interferometers and post-process the experimental data to handle the *π* phase shift due to different initial momentum states. This yields the following local accelerations and acceleration gradients, respectively:

9 $${a}_{j}={a}_{0}-{\Gamma }_{0}{z}_{j}+\frac{1}{2}{\gamma }_{0}{z}_{j}^{2},\qquad {\Gamma }_{j}={\Gamma }_{0}-{\gamma }_{0}{z}_{j}.$$

Inserting those into Eq. 8 and calculating the differential phase between MZI-I and -II yields:

10 $$	\Delta {\phi }_{{\rm{MZI}}}/{k}_{{\rm{eff}}}={\phi }_{{\rm{MZI-II}}}-{\phi }_{{\rm{MZI-I}}} \\=	\left[-\Delta z{\Gamma }_{0}+\frac{1}{2}\Delta z\,\left(\Delta z+2{z}_{0}^{I}\right){\gamma }_{0}\,\right]{T}^{2} \\ 	 -\left[\Delta v\,{\Gamma }_{0}-\left(\Delta z\,({v}_{0}^{I}+{v}_{{\rm{rec}}})-\Delta v\,(\Delta z+{z}_{0}^{I})\right){\gamma }_{0}\,\right]{T}^{3} \\ 	+\frac{7}{12}\Delta z\,{a}_{0}{\gamma }_{0}{T}^{4} \\ 	+{\mathcal{O}}\left({\Gamma }^{2},{\gamma }^{2},\Gamma \gamma \right)$$

with the differential position (velocity) Δ*z* (Δ*v*) between both MZIs.

The butterfly interferometer phase in a quadratic potential is given by ref. ^83^

11 $$\begin{array}{r}{\phi }_{{\rm{BFI}}}/{k}_{{\rm{eff}}}=2\left({v}_{0}+{v}_{{\rm{rec}}}\right)\Gamma {T}^{3}+4a\Gamma {T}^{4}+{\mathcal{O}}\left({\Gamma }^{2}\right).\end{array}$$

Inserting the locally evaluated terms from Eq. 9 into Eq. 11 yields the differential BFI phase:

12 $$	\Delta {\phi }_{{\rm{BFI}}}/{k}_{{\rm{eff}}}={\phi }_{{\rm{BFI-II}}}-{\phi }_{{\rm{BFI-I}} } \\=	\frac{1}{4}\left[\Delta v\,{\Gamma }_{0}-\left(\Delta z\,({v}_{0}^{I}+{v}_{{\rm{rec}}})+\Delta v\,(\Delta z+{z}_{0}^{I})\right){\gamma }_{0}\,\right]{T}^{3} \\ 	 -\frac{1}{4}\Delta z\,{a}_{0}{\gamma }_{0}{T}^{4} \\ 	+{\mathcal{O}}\left({\Gamma }^{2},{\gamma }^{2},\Gamma \gamma \right)$$

where, in contrast to some literature, we used the nomenclature from the main part of our paper where one butterfly interferometer takes a total time of 2*T*.

The differential butterfly with the longest interferometer time 2*T* = 20.5 ms measured a differential phase of $\Delta {\phi }^{{\rm{BFI}}}=0.2{2}_{-0.22}^{+0.1}\,{\rm{rad}}$. As this differential phase result includes zero, we evaluate an upper bound for the acceleration curvature *γ*_0_. For this purpose, we require knowledge about the other terms contributing to the differential BFI phase in Eq. 12. The kinetic properties can be calculated based on our preparation sequence and considering the dynamics determined by Eq. 7. We have quantified the acceleration acting on the *m*_F_ = 2 atoms with a COM time-of-flight measurement as *a*_0_ = (0.11 ± 0.02) m s^−2^. As an approximation for the acceleration gradient, we use the measurement result from the differential MZI with *m*_F_ = 2 atoms, ∣Γ∣ = (39.46 ± 0.02) s^−2^. Taking into account all possible signs of Γ_0_, *γ*_0_, we obtain a maximal region of compatible third-order potential contributions of $| {\gamma }_{0}^{\,\text{ max}\,}| \le 1.73\times 1{0}^{4}\,{{\rm{m}}}^{-1}\,{{\rm{s}}}^{2}$.

We fitted the complete phase model shown in Eq. 10 to our differential MZI data shown in Fig. 4, using as unconstrained parameters both the acceleration gradient Γ_0_ and the acceleration curvature *γ*_0_. Although this complete model is fitting the data well, too, we obtain an inconsistent third-order contribution an order of magnitude larger than our bound ${\gamma }_{0}^{\,\text{ max}\,}$. We thus conclude that the experimental data spread shown in Fig. 5 when using a purely second-order model cannot be sensibly explained by higher-order contributions. To avoid incorrectly fitting unknown effects or drifts in the potential landscape, we refrain from using third-order potential contributions. We make a final simplification to neglect the term $\Delta v \Gamma_0 T^3\, \ll\, \Delta z \Gamma_0 T^2$, being more than two orders of magnitude smaller for our experimental parameters.

## Supplementary information

Supplementary Information

Transparent Peer Review File

## Source data

Source Data

## Data Availability

All NASA CAL raw data used in this study is publicly available through the NASA Physical Sciences Informatics (PSI) website^84^. The processed data shown in the figures and generated in this study are provided in the Source data file. Source data are provided with this paper.
