# Supplementary material for: Magnetometry with a space-based differential atom interferometer
Source: Nat Commun. 2026 Jul 11;17:6089. doi: 10.1038/s41467-026-75230-2 (PMC13356038; doi:10.1038/s41467-026-75230-2)
Supplement: Supplementary file 1 — Supplementary Information [file 41467_2026_75230_MOESM1_ESM.pdf]

Supplementary Information

Magnetometry with a space-based differential atom interferometer

Matthias Meister,[1,a)] Gabriel Müller,[2,a)] Patrick Boegel,[3] Albert Roura,[1] Annie Pichery,[2] David B. Reinhardt,[1] Timothé Estrampes,[2,4] Jannik Ströhle,[3] Enno Giese,[5] Holger Ahlers,[6] Waldemar Herr,[6] Christian Schubert,[6] Éric Charron,[4] Holger Müller,[7] Jason R. Williams,[8] Ernst M. Rasel,[2] Wolfgang P. Schleich,[3,9,10,11] Naceur Gaaloul,[2] and Nicholas P. Bigelow[12]

[1)] *German Aerospace Center (DLR), Institute of Quantum Technologies, Ulm, Germany.*
[2)] *Leibniz University Hannover, Institute of Quantum Optics, QUEST-Leibniz Research School, Hanover, Germany.*
[3)] *Institut für Quantenphysik and Center for Integrated Quantum Science and Technology (IQST), Ulm University, Ulm, Germany.*
[4)] *Université Paris-Saclay, CNRS, Institut des Sciences Moléculaires d'Orsay, Orsay, France.*
[5)] *Technische Universität Darmstadt, Fachbereich Physik, Institut für Angewandte Physik, Darmstadt, Germany.*
[6)] *German Aerospace Center (DLR), Institute for Satellite Geodesy and Inertial Sensing, Hanover, Germany.*
[7)] *Department of Physics, University of California, Berkeley, CA, USA.*
[8)] *Jet Propulsion Laboratory, California Institute of Technology, Pasadena, CA, USA.*
[9)] *Hagler Institute for Advanced Study, Texas A&M University, College Station, TX, USA.*
[10)] *Texas A&M AgriLife Research, Texas A&M University, College Station, TX, USA.*
[11)] *Institute for Quantum Science and Engineering (IQSE), Department of Physics and Astronomy, Texas A&M University, College Station, TX, USA.*
[12)] *Department of Physics and Astronomy, Institute of Optics, Center for Coherence and Quantum Optics, University of Rochester, Rochester, NY, USA.*

(*Corresponding Author. Matthias Meister. matthias.meister@dlr.de)

SUPPLEMENTARY FIGURES

This supplementary material contains additional figures that support the findings presented in the main part of the article.
Supplementary figure S1 shows data of the characterization of the Bragg beam orientation and intensity, which is discussed in detail in the Methods section of the main article.
Supplementary figure S2 shows data of the classical differential center-of-mass motion of the atom clouds during free expansion, which is used to obtain an independent classical measurement of the local magnetic field curvature based on particle motion. The data complements the discussion in the Methods section of the main article.
Supplementary figure S3 shows ellipse data for the differential interferometer campaign with magnetic insensitive $m_{\mathrm{F}} = 0$ atoms as well as data for the differential butterfly interferometer with $m_{\mathrm{F}} = 2$ atoms. The differential phase $\Delta\phi$ obtained from these data sets are used in the main part of the article to determine bounds on non-magnetic forces acting on the atoms as well as higher spatial derivatives of the magnetic field.
Supplementary figure S4 shows histograms of the bootstrap analysis performed to extract confidence bounds for the differential phase of the interferometers. Details on the techniques used here are presented in the Methods section of the main article.

[a)] These authors contributed equally.

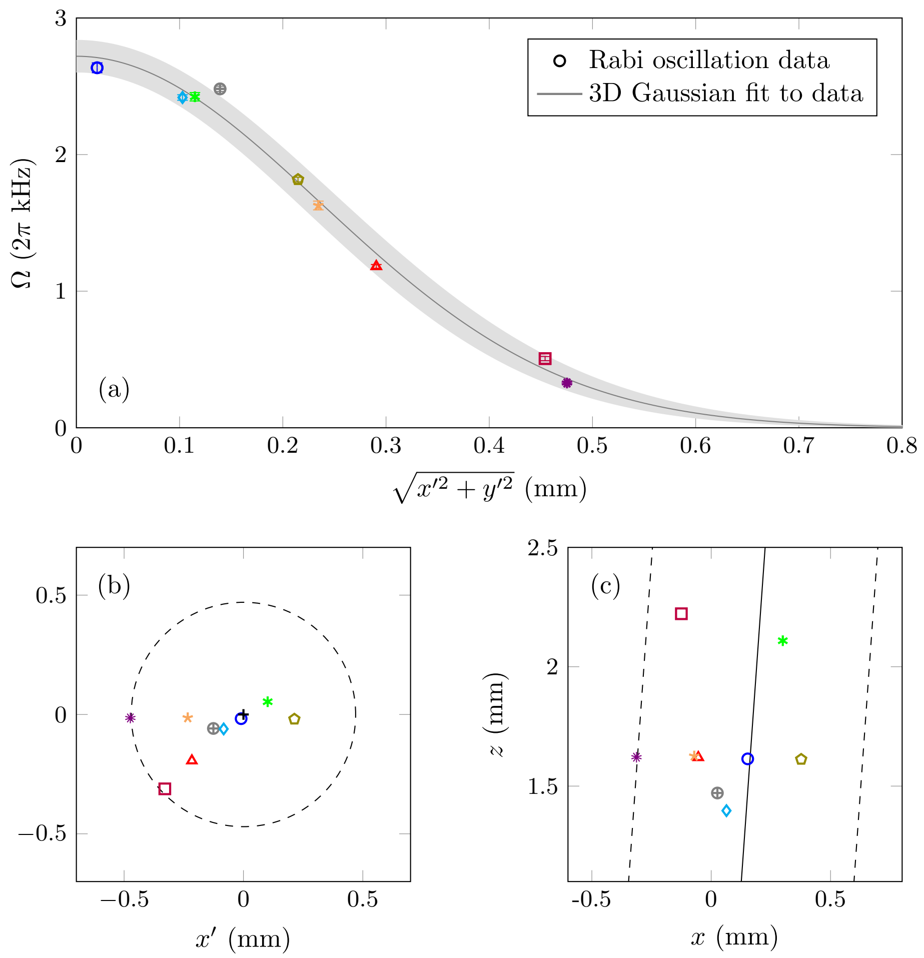

Supplementary Figure S1. **Validation of Bragg beam orientation and intensity:** (a) Rabi frequency Ω of the Bragg beam as a function of the distance from the center of the beam $r' = \sqrt{(x')^2 + (y')^2}$ measured by varying the duration of a single Bragg pulse with atoms at different positions. The error bars correspond to the fit uncertainty of the individual Rabi oscillations (see Methods). A three-dimensional Gaussian intensity profile (gray line and gray shaded area for confidence bounds) is fitted to the measured data points (colored markers) yielding a maximum intensity of the Bragg beam or Rabi frequency of $\Omega_{\text{max}} = 2\pi\,(2.7 \pm 0.1)$ kHz and a beam width of $w = 0.47 \pm 0.02$ mm. (b) Spatial distribution of the individual intensity measurements in the x'-y'-plane corresponding to a view in the direction of the Bragg beam. The origin of the coordinate system marks the center of the beam and the dashed black circle indicates where $r' = w$. (c) Positions of the intensity measurements in the x-z-plane corresponding to the view of Fig. 2a of the main article, where the center of the beam is illustrated by the black solid line and the dashed black lines show where the distance from the center is equal to the beam width. The angle of the Bragg beam with respect to the z-axis was determined to $4.1 \pm 3.9°$ with this campaign. The color and marker style is unique for each measurement point and identical in all three plots.

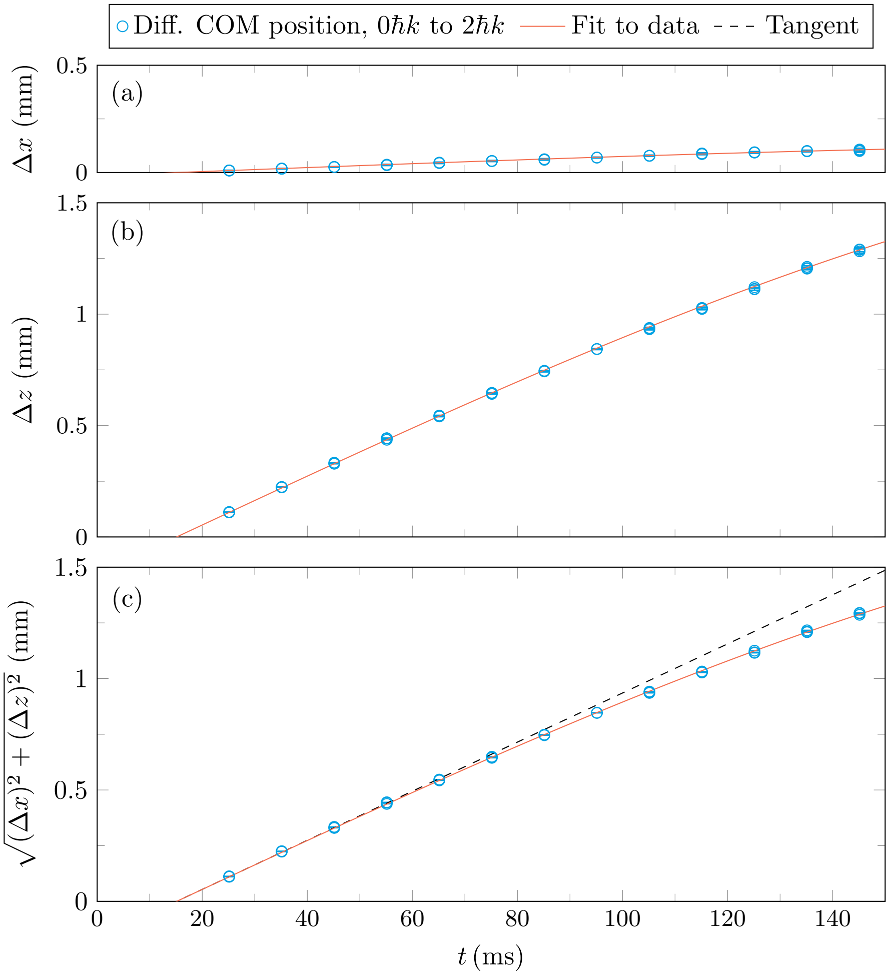

Supplementary Figure S2. **Classical measurement of the residual trap frequency along the Bragg beam by differential center-of-mass (COM) motion:** (a-c) Spatial separation of the $0\hbar$k and $2\hbar$k momentum states of ^{87}Rb BECs simultaneously observed during free evolution up to expansion times of 150 ms. After release from the trap a splitting pulse of the Bragg beam is applied at $t = 15\,$ms for a duration of $0.13\,$ms generating an equal superposition of both momentum states. (a-b) Comparing the distance over time between both states in the x- and z-direction allows determination of the angle $\alpha = (4.77 \pm 0.04)^\circ$ between the Bragg beam and the z-axis. (c) For the total distance the experimental data (blue dots) clearly deviates from a simple linear behavior (black dashed line) due to spatially dependent forces acting on the atoms. Fitting a sine curve (red line) to the data allows to quantify the residual trap frequency along the direction of the Bragg beam to $\omega_{\mathrm{COM},z'} = 2\pi(0.965 \pm 0.014)$ Hz (see Methods).

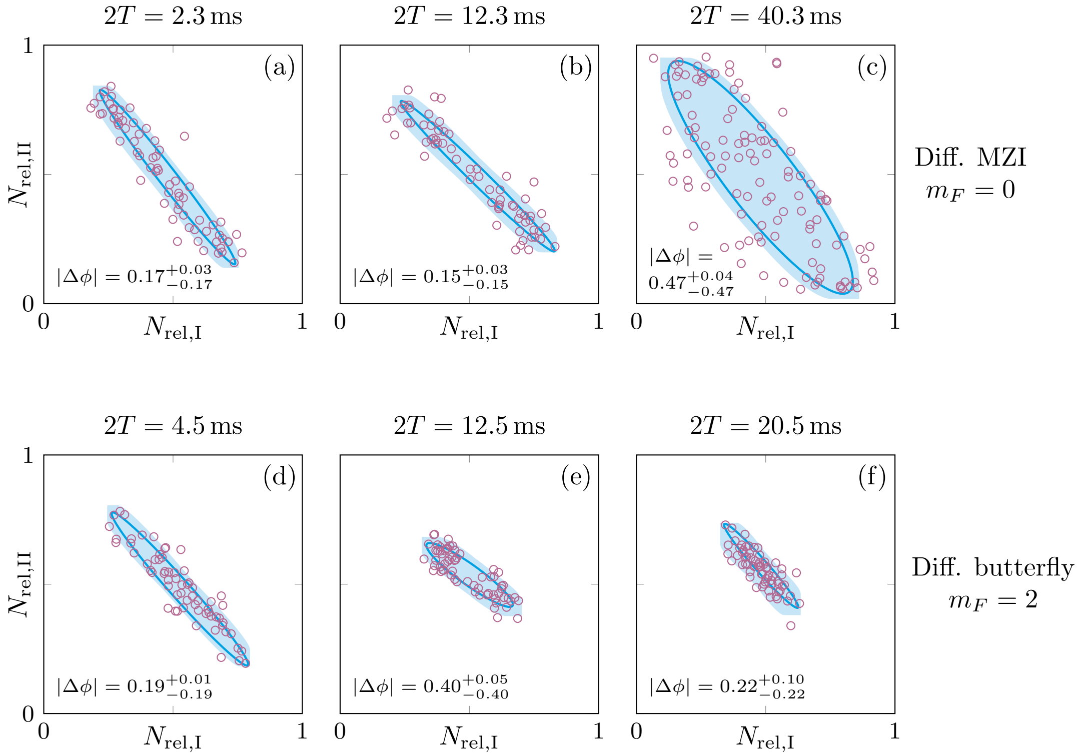

Supplementary Figure S3. **Experimental results of differential Mach-Zehnder and butterfly atom interferometers:** (a-c) Experimental data of the relative populations $N_{\mathrm{rel,I}}$ and $N_{\mathrm{rel,II}}$ in the $0\hbar k$ state of both Mach-Zehnder interferometers MZI I and MZI II described in Fig. 1a of the main article with atoms in the magnetically insensitive $m_{\mathrm{F}} = 0$ state. (d-f) Experimental data of the relative populations $N_{\mathrm{rel,I}}$ and $N_{\mathrm{rel,II}}$ in the $0\hbar k$ state of both butterfly interferometers described in Fig. 1b of the main article with atoms in the magnetically sensitive $m_{\mathrm{F}} = 2$ state. (a-f) In all cases the shape of the correlation data (purple circles) is similar for all interferometer times $2T$ featuring elongated ellipses indicating a differential phase $\Delta\phi = \phi_{\mathrm{II}} - \phi_{\mathrm{I}}$ close to zero obtained by ellipse fitting (blue lines with confidence areas in shaded blue) of the data (see Methods). Although the visibility in the $m_{\mathrm{F}} = 0$ case is quite large even for $2T = 40.3$ ms, the quality of the correlation data diminishes due to detection noise becoming an increasing issue for long expansion times with a small total atom number.

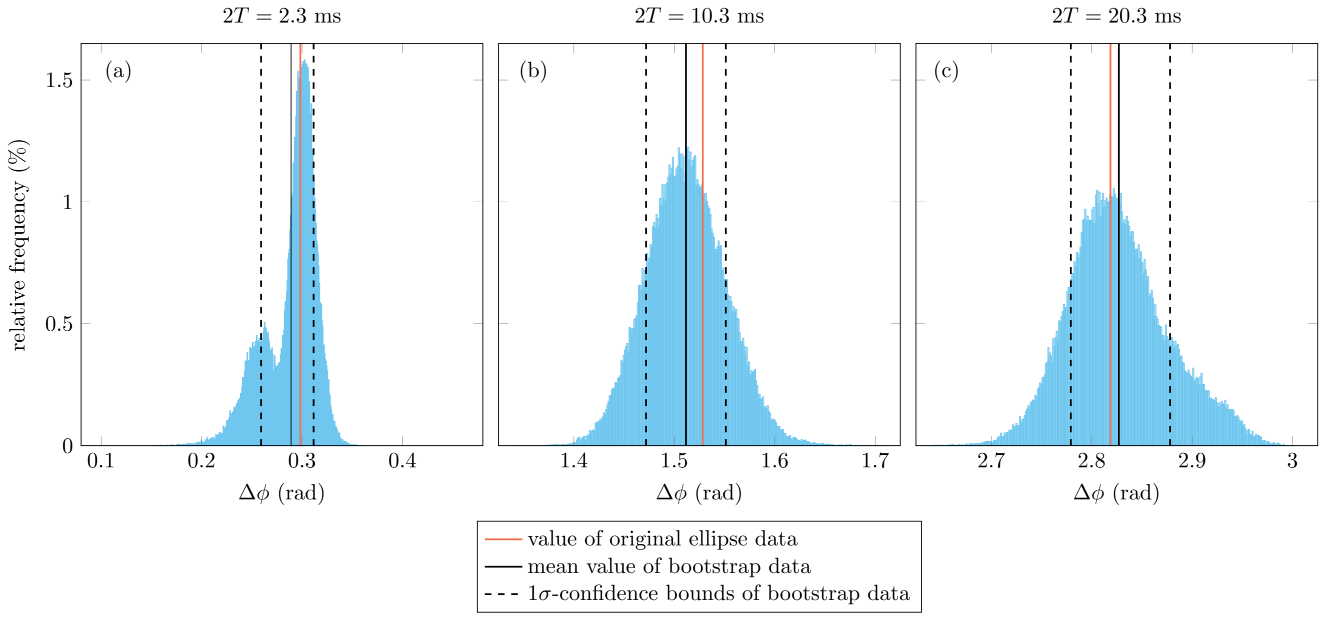

Supplementary Figure S4. **Bootstrap analysis of the differential phase for differential Mach-Zehnder atom interferometers:** (a-c) Histograms of the differential phase $\Delta\phi$ obtained by fitting a total of 10^5 bootstrapped data sets sampled from the corresponding experimental data sets shown in Fig. 3a-c of the main article for interferometer times $2T = 2.3$ ms, 10.3 ms, and 20.3 ms (see Methods). The value of $\Delta\phi$ obtained from the original experimental data set (red line) is in all three cases close to the mean value of the bootstrap histogram (black line) indicating the high consistency of the data sets. The 1σ-confidence bounds (black dashed line) are considered as the uncertainty of the phase estimation. The histogram in subplot (a) features a bimodal distribution which is caused by the few outliers with very high or low measured population displayed in Fig. 3a, while the histograms in subplot (b) and (c) approximately follow the typical Gaussian behavior.
