# Supplementary material for: Magnetometry with a space-based differential atom interferometer
Source: Nat Commun. 2026 Jul 11;17:6089. doi: 10.1038/s41467-026-75230-2 (PMC13356038; doi:10.1038/s41467-026-75230-2)
Supplement: Supplementary file 2 — Transparent Peer Review File [file 41467_2026_75230_MOESM2_ESM.pdf]

nature portfolio

Peer Review File

Magnetometry with a space-based differential atom interferometer

Corresponding Author: Dr Matthias Meister

This file contains all reviewer reports in order by version, followed by all author rebuttals in order by version.

Version 0:

Reviewer comments:

Reviewer #1

(Remarks to the Author)
The paper by Meister et al. reports on the successful demonstration of a BEC and its application for magnetic curvature measurements.
The special trick here is that this is all done in space, in a huge collaborative effort around NASA's Cold Atom Lab (CAL) aboard the ISS.
This is an impressive technological feat and as such already interesting for the global readership of Nature Communications.
The data are trustworthy and the paper is technically sound.

There is no doubt to me, that the paper should be published, and also that NCOMMs is a good place for it.
However, the presentation will clearly profit from a number of refinements as outlined below:

This work shows a significant advance in space-based quantum sensing:
• It demonstrates that differential atom interferometry can work in the noisy ISS environment. It will be even better in a dedicated quantum sensing satellite one day.
• It demonstrates magnetometry and validates the magnetic environment for future gravity measurements aboard ISS (if they can be done during the expected life time of ISS).
• The differential measurements successfully suppress common-mode noise (laser phase, vibrations)
• The paper prepares and supports upcoming missions like BECCAL and provides crucial lessons for planned fundamental physics experiments in space (AEDGE, STE-QUEST)

The key achievements include:
• A measured magnetic field curvature inside the ISS vacuum chamber: B'' = 620 nT/mm^2 using differential atom interferometry. Individual measurements are as accurate as about 10 nT /mm^2
• They have extended the interferometer coherence time to 40.3 ms, which is 10x longer than previous space demonstrations, however, still more than an order of magnitude shorter than in a drop tower or fountain on Earth. There is still room for improvement as it is the very purpose of going to space to have reduced perturbations and longer coherence times.
• They were able to discriminate magnetic from non-magnetic effects by comparing magnetically sensitive (mF = 2) and insensitive (mF = 0) states.
• On the technical side, they achieved 85% Bragg pulse efficiency and ultra-low BEC expansion energies (0.66 nK), which promises longer coherence times in the future.
And even though one tends to get used to such highlights, it really noteworthy and I do admire energies as low as 660 pK. To get this done without major human intervention in a lab that is flying even over my head typically a few times per day ... is kind of stunning.

The paper is comprehensive (and rather technical) and the non-specialist will need more guidance to the key results.

The introduction starts very general, which is a good thing, but then it appears to be mixing magnetometry that is relevant to calibrate accelerations in future atom interferometry in space and applications where geophysical parameters should be

measured. It appears to me that these two different applications will eventually be realized by two entirely different types of sensors as the boundary conditions and required accuracy and time resolution limits are vastly different. It might be useful just to add one sentence to say that quantum sensors in space are important but also say that the solution presented here solves a specific set of tasks.

The RESULTS section hits the innocent reader really hard from the start with technical details (not results) that are only relevant to those who build and use the system.
Already line 5 is the "switch off" button for non-specialists, when it says in the introduction to the results:

"This final trap is aligned with the Bragg beam at a position of (x,y, z)=(−0.07, −0.11, 1.04)mm with trap frequencies (ωx,ωy,ωz) = 2π ×(9.28, 21.2, 18.1) Hz."

No one needs to know the position of an atom cloud of undefined diameter in an undefined reference frame. The reader wants to know

a) what the grand goal of the experiment is
b) that you start with cold atoms
c) that you get a BEC
d) that you have an atom interferometer and which type of interferometer
e) how you read the phases and how stably you can do this
f) how the magnetic fields influence this entire setting.
g) why this is well done on the ISS

If this entirety can be presented as figure 1, this would be the dream figure 1.
In its present form, Figure 1 is of very moderate use. The chip indicates some wires, which are needed to make the cold atoms… but they are not described in the text not key in understanding of the paper. The story about the tilt of the Bragg beam seems to be important to the team, but the general reader will find this too technical. The story about the angle of the laser beam finds a perfect place in the methods.

For non-specialists it is most important to see what atom interferometry is about. And Figure 2 is very nicely done.
One may also substitute Figure 1 by Figure 2.

Since the paper is about an experiment aboard the ISS, the general reader would expect some graphical hint of how the experiment is fit on the ISS. For NCOMMS I think this would be a strong plus, to show how and where the experiment is embedded into the space station, maybe even to known scales. It would also be good to know what has been done to eliminate vibrations, which are still omnipresent because of people and machines. Such or similar figures have existed in N-Journal derivates but I think it is still the key to why it must be published in NCOMMS.

Many paragraphs of the "results" section should move to the “methods” without any non-specialist missing them:
Almost all of
o line 135 – 154
o line 328 – 229
o line 358 – 399

Additional information and questions may come up instead:

Naively one might think that the orientation to the precessing motion of the ISS is relevant. Is it?

Magnetic field gradients exist in all directions. How can the vector character be probed in the interferometer?

While the idea of going to a zero-g environment is that perturbations go down and coherence times up, achieving this goal is still challenging. On the ground, on can have more atoms, better shielding, better vibration isolation, longer coherence time (than so far in space). In the outlook it would be interesting to see a 2-4 sentence timeline how the obstacles (everybody understands that they must be there at the start) can be overcome. This must be presented to NASA anyway.

The field curvature is given as |B′′| = (622.08 ± 19.66) nT mm^2. Given the error bar, it is probably more adequate to write this as 620 ± 20 nT/mm^2.

t would be helpful to have a comparison: the best ground based optically pumped magnetometers can measure fT fields, but certainly not two in 1 mm distance. But how does all that compare and what is the cause for the curvature. What would be its time dependence? How much of that is locally generated by the atom chip ? How much is ISS noise?

And why is B curvature more relevant than B or grad B ? Do they get these numbers also and if so with which accuracy ? Normally this is what the standard metrologist would search for.
Being capable of seeing the curvature is a great thing. But again: start from a common ground.

The ellipse fitting is a very useful tool in a noisy interferometer environment and I understand that the authors feel this should by now be known by everyone. But it is not.
So before talking about bootstrapping algorithms (which I could not follow in the main text) they should remind us (evtl. also

with a reference) why this helps seeing contrast even when a single interferometer alone is dephased. NCOMMS is also read by chemists and geodesist.

The system is currently still limited by atom number (3×10^3 atoms per exit port) which constrains sensitivity and noise. What are the prospects for eventually pushing this by 3 orders of magnitude?

The achieved sensitivity still needs a factor of 10x to get to the shot-noise limit. So even without growing the number of atoms in the BEC: what can be done ?

Summarizing:

Yes the work should be published by Nature Communications. There is no need to generate new data or data processing. Being capable of doing such experiments under such circumstances is a great feat and I am certain that also an interdisciplinary community will enjoy that this can be done.

However, it will be good and important to see a second re-organized and re-worded version that is communicating with the interdisciplinary community.
This can certainly be prepared rather fast, maybe initially by a single one of the senior authors (who have done such restructuring many times before) before it is re-submitted for (most likely acceptance) by Nature Communications.

Reviewer #2

(Remarks to the Author)
The manuscript introduces methods to measure magnetic field and its higher spatial derivatives in a localized volume aboard the International Space Station (ISS) using light-pulse atom interferometry (LPAI) of Rubidium-87 (Rb-87) Bose-Einstein Condensates (BECs). The reported data represents the latest measurement campaign of the Cold Atom Laboratory (CAL), advancing from prior works (Refs. [45,46]) utilizing Bragg LPAI, which was made possible after the Science Module 3 (SM-3) upgrade to CAL. A key differentiator of this work is the implementation of differential LPAI measurements to enable overcoming ISS vibrations and thereby extending interrogation times (2T) to ~40 ms. Magnetic field measurements employ two differential LPAI geometries–Mach-Zehnder Interferometer (MZI) and Butterfly Interferometer (BFI)–and two magnetic sublevels of Rb-87 (m_F = 0, 2). The differential MZI and non-interferometric atom cloud differential center-of-mass (COM) measurements provide complementary measurements of the magnetic field curvature (~600 nT/mm^2) and the BFI is used to constrain the third spatial derivative of the magnetic field.

The manuscript is well-written and sufficient details regarding data analysis are presented in the methods section. Empirical results from the differential LPAI are mostly convincing (more on that below). However, I have reservations about whether the scope and impact of this work meet the standards of Nature Communications. Therefore, I invite the authors to address the following critiques.

Based on the information contained in the manuscript, this work doesn't appear to be a sufficiently big technical or scientific leap compared to the previous two publications Ref. [46] and Ref. [45] by CAL in Nature and Nature Communications respectively. No hardware upgrades were required. The changes in the interferometer geometries appear to be the result of straightforward reprogramming. The scientific pitch is a bit stronger. As evident from the manuscript title, this work's scientific motivation presents a pivot from Ref. [45], which focused its science motivation on gravitational physics. This manuscript's introduction provides a strong motivation (e.g. geophysics) for in-orbit magnetic field sensing. Moreover, the authors adequately justify the advantage of localization of magnetic field measurements compared to equivalent Earth-based LPAI sensors. However, I am skeptical about LPAI being the most appealing platform for space-based magnetometry. Given the diverse readership of Nature Communications, the manuscript would more effectively sell the LPAI as a magnetic field sensor if *quantitative* comparison to non-LPAI platforms were presented. Even if current specs are not competitive with non-LPAI platforms, I recommend arguing a niche that only LPAI magnetometers can fill in future missions.

My recommendation to publish in Nature Communications is contingent upon satisfactory responses to the above points.

Irrespective of the verdict of this submission, I think this work is of value to the microgravity LPAI community and might inform future missions like "BEC and CAL" (BECCAL). Hence, I believe publication (irrespective of location) is warranted after some revisions. In the following, I will identify possible avenues for strengthening the manuscript, elaborate on concerns about some of the measurements and list technical questions/comments which might be of relevance for future work.

1. In the conclusions, the authors' comment about Delta-Kick Cooling (DKC) being unnecessary (lines 260-262) appears to contradict an earlier statement (lines 147-148) that cloud expansion is one of the two factors limiting the visibility. Please reconcile those statements in the revised manuscript.

On a related note, is DKC possible with the redesigned atom chip of SM-3? I didn't find any mentions of DKC capability in Refs. [45,46]. Moreover, in the methods section of Ref. [47], it says:

"Later on, the core part of the apparatus, including the atom chip, was exchanged leading to a new magnetic field geometry and thus preventing the further continuation of the experiments reported in this article."

Was this because the Z-, H-, U-traces were moved further apart to accommodate the Bragg beam? I leave it optional to elaborate on this in the manuscript if the authors are struggling with space constraints. Though, I encourage the authors to include these details in other publications.

2. The manuscript highlights limitations on 2T to 24.3 ms for the m_F = 2 interferometers (lines 147-148 & 164-166) arising from transverse COM motion of the atom cloud (and cloud expansion) relative to the Bragg beam center due to accelerations from residual magnetic field gradients. BECCAL is supposed to have a piezo actuated tip-tilt mirror for rotation compensation. How much visibility improvement can be obtained by utilizing this feature to center the beam on the atom cloud? Alternatively, could the transverse atom cloud motion be converted from a bug to a feature by using the control signals from the rotation compensation system to tease out transverse magnetic fields?

3. Aside from rotation compensation, could visibility be improved by using quantum optimal control? I understand that CAL's current hardware has a limit on time resolution (~10 μs) for amplitude control of atom optics pulses. So, generating non-square pulse shapes might be challenging. What is the temporal phase resolution? Decent phase control would permit adiabatic rapid passage (ARP) on the Bragg beams. I only found discussion of ARP in the context of radio frequency transfer between m_F = 0 and 2. There are many examples in the literature improving pulse transfer efficiency via phase-only quantum control. Could the authors exploit such schemes to improve pulse transfer efficiency beyond the current 85%?

4. With the information provided in the manuscript, empirical support for judging the veracity of the BFI results is inadequate. This is especially important given that the conclusion is a null result on the third spatial derivative of the magnetic field. This is in stark contrast to the differential MZI which is corroborated with an independent non-interferometric differential COM motion measurement of the atom clouds. As a proof-of-principle, can the authors inject a fake third spatial derivative of the magnetic field signal to build confidence in the differential BFI? If the current hardware doesn't support injection or the mission schedule is too restrictive, I recommend including as much additional theoretical support and/or additional data as possible for the interpretation of the differential BFI results. For example, a skeptical reader would benefit from augmenting Fig. 3d to include visibility data from BFI-I and BFI-II.

5. Minor point: Ref. [36] is a review article on the superconducting gravimeter. Given this manuscript's focus on magnetism, it would make more sense to cite a standard reference on Superconducting Quantum Interference Device (SQUID) magnetometers.

Reviewer #3

(Remarks to the Author)
Review of NCOMMS-25-82843-T "Space magnetometry with a differential atom interferometer"

The manuscript is based on atomic interferometer located in space. Here they report on measurement of magnetic fields gradients and curvature by comparing m=2 (magnetic sensitive) to m=0 (magnetic insensitive) states using a differential atomic interferometers. The differential geometry largely suppresses the sensitivity to laser phase noises and hence allows tens of msec interrogation times eventually limited mostly by cloud expansion and the finite size of the laser beams (where the record low sub nano-K effective temperature of eth expending cloud helps a lot)
The results show high sensitivity to magnetic field gradients, lack of the effect for magnetic field insensitive transition (verifying the do measure magnetic field related effects) and lack of magnetic field curvature measured with a so called butterfly geometry that suppresses eth sensitivity to constant gradients
The magnetic field they measure is some local artifact of their system and is not a priority known, so they accuracy of their method is not clear. It is estimated by comparing the AI measured B gradient to the crudely measured one by observing the COM motion of the atoms (with a ~3 sigma discrepancy, discusses around line 229)
The manuscript is clear, and technically sound. However I have some concerns about its novelty and impact
Several manuscripts were already published by the same group describing earlier results and establishing the basic operation of their system. Indeed the ability to carry such complicated and sensitive experiment in space is extremely impressive and does open exciting possibilities for future high impact experiments in 2 aspects: first measuring effects such as gravitational waves and planetary missions where space is inherently needed and second: micro gravity can significantly improve interrogation times (especially with the super cold atomic source they use) and also improve compactness and hence localization of the measurement
In the current manuscript the measurement is not important by itself but rather a demonstration and (partial) validation of another technical aspect: measurement the residual magnetic gradient that will be important for future precision measurement
Moreover the technique used is similar to previous atomic gradiometers demonstrated on earth
Finaly the quality of the measurement is not impressive on absolute scale (again the ability to carry such experiments on space is indeed impressive): interrogation times are rather small (probably due to the sub mm diameter of their laser beams where on earth people routinely use several cm beams and demonstrate 100 times longer interrogation). Also their signal to noise is quiet poor as compared to many earth experiments due to the small number of atoms they use.
To conclude: while in general the pursuit of atomic interferometers in space is highly motivated, advancing well and has many exciting prospects I find the current manuscript describing another technical step without strong innovation in methods or results and while clearly needed for future measurements is only marginally providing the wide impact needed for nature com

Few technical comments (line numbers in brackets):

1. (62) "This allowed the execution of various differential interferometer geometries up to total interrogation times of 2T = 40.3ms..." considering this is important part of the paper novelty - there should be an elaborate explanation of how they achieved this. As I understand it, differential interferometry isn't enough to overcome their previous limitation.
2. (68-69) The bound on non-magnetic acceleration gradient is expressed strangely, it's sufficient to say that they've bound it below 0.3 s^-2. Honestly it's an insignificant result of this work and holds value merely as a sanity-check. I would omit it from the introduction entirely.
3. (111) It should be made clear that this acceleration gradient measurement is classical with no interferometry involved.
4. (140) There's a phase ambiguity when the 2 interferometers are in-phase. A frequency shifted mirror pulse can add an artificial phase shift between the clouds and solve this issue (PRL 118, 160401 (2017)). If this solution is prohibitive in CAL, it's worthwhile to mention it.
5. In general precise measurement of the in-chamber magnetic gradient isn't motivated well enough

Version 1:

Reviewer comments:

Reviewer #1

(Remarks to the Author)
The authors have integrated a large part of the comments by all referees and responded to the open questions and also optimized the figures. I suggest acceptance and advancing to the next stage towards publication.
The only things the authors may want to consider along the way – without me needing to see the manuscript another time:

• Title:
“Space magnetometry with a differential atom interferometer”
This reads as if the atom interferometer measured the B-field distribution of space, like Voyager travelling through the solar system. However, the measurements rather concern the very local field which needs to be known for instance as a background for WEP measurements later.
Probably more correct: “Magnetometry with a space-based differential atom interferometer” ?
• Punctuation and orthography
o Line 53: Single Bragg -> single Bragg ?
o Line 63: “contributions.. “ only one “.”
But these things will be sorted out during the editing process, anyway.
• Vibration unit
“order of 10−9g^2/Hz” is technically correct, but may sound unusual for a general reader, since it gives the acceleration power spectral density, not an acceleration. I suggest to give the acceleration amplitude, in a frequency band of 1 Hz and some information about the spectral variation: is this the max value at about 10-100 Hz of noise, which is the most relevant range for the measurements?
• Punchline of the introduction

a) The authors want to make a double statement:
1. The have improved numerous parts of the experiment and overcome technical limitations of previous experiments in CAL.
2. Based on that they have performed in situ magnetometry and obtain interesting values on gradients and curvatures.
This is somehow captured in line 49, but there is an opportunity to formulate things more positively, by deleting line 47 - 50 and simply continuing with the improvements described starting with line 51.
b) Their lines 64-68
We foresee two different applications of the magnetic curvature sensor in space, the first one targeting the in-vacuum characterization of magnetic fields in aforementioned fundamental physics experiments based on atom interferometry. In such experiments, for instance, when testing general relativity8–10, one of the leading systematic effects coupling into the signal are magnetic field gradients. Fully avoiding such couplings is not possible, neither by operating on a magnetically insensitive sub-level due to the non-linear Zeeman effect, nor by advanced compensation techniques39. Thus, magnetic field gradients need to be mapped microscopically at the measurement location, i.e., inside the vacuum chamber where only the atoms have direct access. Devices such as vector fluxgate magnetometers and scalar atomic magnetometers generally offer high precision, however, they have to measure from out-of-vacuum, naturally impeding the performance for gradient reconstruction at the relevant location, especially considering higher order spatial components. In contrast, utilizing a differential atom interferometric magnetometer enables precise in-vacuum characterization of magnetic field gradients at the right spot. Multiple orders can be mapped out by deploying the differential interferometer at various positions or by adapting the scheme to be sensitive to a different order, as presented in this work. Due to the absence of gravity, performing such characterizations in orbit instead of during an on-ground commissioning phase provides the advantage of more localized measurements under the true environmental conditions.

Could be simplified and presented more:
We foresee two different applications of the magnetic curvature sensor in space: first the in-vacuum characterization of magnetic fields for tests of general relativity, where one of the leading systematic effects is due to magnetic field gradients. Thus, magnetic field gradients need to be mapped microscopically at the measurement location, i.e., inside the vacuum chamber where only the atoms have direct access. Fluxgate magnetometers and atomic magnetometers can offer higher precision, however, they measure from the outside, impeding the reconstruction of gradient and higher order derivatives of the magnetic field. A characterization in orbit is free of the influence of gravity, allowing for more localized measurements

under the experimental conditions of future gravity experiments.

One may find some more paragraphs where the key message can be conveyed more clearly simply by reducing or eliminating parts of sentences and avoiding repetition.
Line 91
We perform two types of differential atom interferometer geometries

Is it grammatically ok to “perform a geometry” ? or would you want to say: We have realized differential atom interferometry in two geometries.
Tenses
The text occasionally change tenses between different paragraphs. It feels a bit uneven…but the editing team will go through grammar and style during the final process, anyway.

I am otherwise happy with the manuscript and graphs.

Reviewer #2

(Remarks to the Author)
Recommendation: Accept

I thank the authors for their detailed responses to my comments and thoughtful revisions to the manuscript. In particular, I appreciate the new visibility data and correlation plots for the Butterfly Interferometer as well as the expanded discussion of center-of-mass dynamics and cloud expansion. The changes have substantially strengthened both the accessibility and made a stronger case for the scientific impact of the work.

Reviewer 1's emphasis on broader readability prompted valuable structural improvements that benefit the interdisciplinary readership of Nature Communications. My own initial reservations—shared in spirit with Reviewer 3—centered on whether this work represented a sufficient advance over prior high-impact publications by the Cold Atom Laboratory (CAL). However, the authors have alleviated my concerns for the following reasons.

The authors have satisfactorily addressed my critiques by better contextualizing their work within the landscape of space-based magnetometry and articulated a unique niche for light-pulse atom interferometry (LPAI). Specifically, the quantitative comparisons between LPAI shot noise projections and established sensors—such as fluxgate and optically pumped magnetometers—highlight the competitiveness and superior spatial resolution of the LPAI approach. Furthermore, while addressing Reviewer 1, the authors outline a credible strategy for achieving shot-noise-limited performance in dedicated future space missions that presumably won't share the limitations of a multi-user facility like the International Space Station (ISS). By reframing their sensor as "multi-modal," the authors have convincingly argued for filling a niche in geophysics and fundamental physics (e.g., tests of general relativity) research.

Therefore, I recommend acceptance of the manuscript in its current form. No further revisions are required.

Reviewer #3

(Remarks to the Author)
I carefully read the author's answers to all referees comments and the revised manuscript.
In their answers to my comments the authors stressed eth future prospects enabled by the further advance presented in the current manuscript over their previous works. I do accept these prospects but am still debating whether they justify publication in a high profile general readership publication.

The changes in the manuscript do address all technical comments by all referees and do make it more comprehensive and a bit better motivated by do not fundamentally change its impact and novelty.

Open Access This Peer Review File is licensed under a Creative Commons Attribution 4.0 International License, which permits use, sharing, adaptation, distribution and reproduction in any medium or format, as long as you give appropriate credit to the original author(s) and the source, provide a link to the Creative Commons license, and indicate if changes were made.
In cases where reviewers are anonymous, credit should be given to 'Anonymous Referee' and the source.
The images or other third party material in this Peer Review File are included in the article’s Creative Commons license, unless indicated otherwise in a credit line to the material. If material is not included in the article’s Creative Commons license and your intended use is not permitted by statutory regulation or exceeds the permitted use, you will need to obtain permission directly from the copyright holder.
To view a copy of this license, visit https://creativecommons.org/licenses/by/4.0/

Author response to referee comments

Manuscript "Space magnetometry with a differential atom interferometer" NCOMMS-25-82843-T

Reviewer 1

We thank the reviewer for their positive assessment of our manuscript as well as for their recommendation for publication in *Nature Communications*. We are especially grateful for their dedicated and detailed feedback regarding several avenues for refining our message, which helped us substantially in making our work more accessible for the broad readership of *Nature Communications*.

In particular, we would like to highlight the adaptions we've made in the Introduction to now clearly describe the two separate applications of our method that might also speak to two different audiences, as well as an added comparison to optically pumped magnetometers and further clarification of enhancements recommended for future atom interferometry space missions.

In the following, we provide a point-by-point response to the reviewer's comments.

Reviewer Comment 1.1 — The introduction starts very general, which is a good thing, but then it appears to be mixing magnetometry that is relevant to calibrate accelerations in future atom interferometry in space and applications where geophysical parameters should be measured. It appears to me that these two different applications will eventually be realized by two entirely different types of sensors as the boundary conditions and required accuracy and time resolution limits are vastly different. It might be useful just to add one sentence to say that quantum sensors in space are important but also say that the solution presented here solves a specific set of tasks.

Reply: We thank the reviewer for this important feedback on how to advertise our proposed measurement technique in a clear and concise way. We fully agree that the way we introduced the two different applications, first, for in-vacuum magnetic field characterization and second, for the measurement of geophysical parameters, their separation in terms of sensor and platform requirements was not clear.

Indeed, for the first application the main mission design driver is the fundamental physics measurement itself, our atom interferometer based magnetic sensor merely has the advantage compared to alternative magnetometers that it can operate exactly where, finally, the mission-critical measurements will be performed. By contrast, the application as a sensor for geophysical parameters relies on a dedicated experiment and platform design to allow for the measurement of external fields to the specified level. Additionally, as only mentioned in the discussion section before, our sensor would also allow to measure non-magnetic forces such as gravity gradients with only slight adaptions of the scheme.

To make this important distinction, we restructured our *Introduction* to first explain the general measurement we perform and then provide two clearly separated paragraphs with discussions of the two proposed applications.

Reviewer Comment 1.2 — The RESULTS section hits the innocent reader really hard from the start with technical details (not results) that are only relevant to those who build and use the system. Already line 5 is the "switch off" button for non-specialists, when it says in the introduction to the results:

This final trap is aligned with the Bragg beam at a position of $(x, y, z) = (-0.07, -0.11, 1.04)$ mm with trap frequencies $(\omega_x, \omega_y, \omega_z) = 2\pi \times (9.28, 21.2, 18.1)$ Hz.

No one needs to know the position of an atom cloud of undefined diameter in an undefined reference frame. The reader wants to know

a) what the grand goal of the experiment is b) that you start with cold atoms c) that you get a BEC d) that you have an atom interferometer and which type of interferometer e) how you read the phases and how stably you can do this f) how the magnetic fields influence this entire setting. g) why this is well done on the ISS

If this entirety can be presented as figure 1, this would be the dream figure 1. In its present form, Figure 1 is of very moderate use. The chip indicates some wires, which are needed to make the cold atoms… but they are not described in the text not key in understanding of the paper. The story about the tilt of the Bragg beam seems to be important to the team, but the general reader will find this too technical. The story about the angle of the laser beam finds a perfect place in the methods.

For non-specialists it is most important to see what atom interferometry is about. And Figure 2 is very nicely done. One may also substitute Figure 1 by Figure 2.

Reply: We thank the reviewer for their valuable feedback about the beginning of the results section and the arrangement of Figures 1 and 2. In order to make the paper more accessible for a broader readership, we have done the following:

(i) We added a new paragraph directly at the start of the results section to explain the grand goal of the experiment and the key steps required to fulfill it. This includes referring to the interferometer sequences, explaining that cold atoms in a BEC are the basis of our experiment and what and how we perform the measurement. The paragraph is of course not very detailed, since all topics are discussed in more detail later, but we think that this should help to understand what the paper is about in a nutshell.

(ii) We switched the order of Figures 1 and 2 such that the general concept of the atom interferometer is the first visual impression the reader sees. It is therefore already mentioned in the initial paragraph.

(iii) We added two new subplots to the Figure illustrating the atom chip and beam properties (now Figure 2) such that there is more quantitative value in it. Fig. 2c shows the motion of the atoms orthogonal to the Bragg beam indicating that the atoms move transversal to the beam in a substantial manner and thus reducing the efficiency of the interferometer pulses. Fig. 2d shows the expansion dynamics of the BEC in all three spatial directions and thus gives a good impression of the size of the atom clouds as well as their effective temperature.

(iii) We adjusted section II.A of the results to start with a discussion of the location of the experiment onboard the ISS (see next reviewer comment).

(iv) We removed the specification of the trap position and uncertainties of the initial velocities to keep the first part of the results section less technical as suggested by the reviewer.

(v) The process of how the phases are determined from the measurement data is now explained more accessible in section II.B when the interferometers are discussed in detail.

Reviewer Comment 1.3 — Since the paper is about an experiment aboard the ISS, the general reader would expect some graphical hint of how the experiment is fit on the ISS. For NCOMMS I think this would be a strong plus, to show how and where the experiment is embedded into the space station, maybe even to known scales. It would also be good to know what has been done to eliminate vibrations, which are still omnipresent because of people and machines. Such or similar

figures have existed in N-Journal derivates but I think it is still the key to why it must be published in NCOMMS.

Reply: The general setup of CAL and how it is installed aboard the ISS have been covered by references [1–3] in detail. Following the reviewer's general advice, we have added an explanation of the location and arrangement of CAL at the beginning of the Results section to give some information about the platform including a statement about the vibrations. To leave graphical space for other important aspects of our results, we believe it is more beneficial to refer the reader to the above references for details on CAL.

Specific vibration isolation is not possible for CAL since the machine is quite heavy (> 200 kg) and suitable vibration isolation cannot be integrated in the EXPRESS rack due to size and weight limitations. A detailed analysis of the ISS vibrations and their impact on atom interferometry is done in reference [3]. To overcome the limitations due to vibrations we have performed differential interferometer campaigns to cancel out common phase contributions, which we describe in detail in the main part of the paper. For the interest of future missions that require non-differential measurements, we suggest the exploitation of a vibration isolation scheme giving a reference for the interested reader [4].

Reviewer Comment 1.4 — Many paragraphs of the "results" section should move to the "methods" without any non-specialist missing them: Almost all of line 135 – 154, line 328 – 229, and line 358 – 399

Reply: We thank the reviewer for their feedback and have tried to accommodate the respective changes. Most of lines 135-154 have been moved to the "ellipse fitting" section of the Methods.

Unfortunately, the second statement is unclear (line 328 – 229). Lines 228-229 would be part of the discussion section, but contain information that we see appropriate for a discussion. We assume the reviewer refers to lines 328-339 instead which discuss the analysis of absorption imaging data. These are part of the Methods already and we did not change them. Similarly, lines 358-399 discuss the ellipse fitting and are part of the Methods and were not changed.

Reviewer Comment 1.5 — Additional information and questions may come up instead:

Naively one might think that the orientation to the precessing motion of the ISS is relevant. Is it?

Reply: Indeed, the rotation of the ISS while orbiting Earth can have an influence on the experiment performance of atom interferometers. However, on the time scales accessible in the current implementation they generally play a negligible role.

In particular, the precessing motion of the ISS, i.e., the one about the Bragg beam axis, does not enter the phase of our interferometer due to its alignment with the sensitive interferometer axis, assuming to first order $\vec{k}_{\text{eff}} \,||\, \vec{e}_z$. This can be directly seen by calculating the non-differential interferometer phase sensitive to Coriolis effects $\phi = 2\vec{\Omega} \cdot \left(\vec{v}_0 \times \vec{k}_{\text{eff}}\right) T^2$ [5].

In addition to this precessing motion, the ISS rotates once per orbit to keep its nadir pointing towards Earth. This results in an angular velocity component orthogonal to the effective wave vector $\vec{k}_{\text{eff}}$, i.e., $\Omega_y \approx 1\,\text{mrad}\,\text{s}^{-1}$. According to the above phase relation, the non-vanishing term scales as $2\Omega_y v_x k_{\text{eff}}$ in a non-differential Mach-Zehnder configuration. For $T = 20\,\text{ms}$ and $v_x = 3\,\text{mm}\,\text{s}^{-1}$, this would result in a phase shift of $\approx 2\,\text{mrad}$ which lies well below our phase resolution. The differential MZI even only measures a differential phase scaling with Δv_x between the respective MZI input states which should be zero (or very close to zero) in our measurement scheme and thus not influence our measured phase.

Reviewer Comment 1.6 — Magnetic field gradients exist in all directions. How can the vector character be probed in the interferometer?

Reply: Assuming a single fixed Bragg beam orientation $\vec{e}_{z'}$, a single MZI measures the component of an acceleration $a_{z'}$ that is aligned with the Bragg beam. Here, we consider the potential $V = m_F\, g_F\, \mu_B |\vec{B}|$ due to the linear Zeeman shift. Thus, the measured acceleration corresponds to the spatial derivative along z' of the absolute magnetic field via $a_{z'} \propto \partial_{z'} |\vec{B}| \equiv B'_{z'}$. In order to probe other magnetic field gradients, one would need a multi-axis Bragg beam setup, where a MZI driven by an individual beam measures the gradient along that given beam axis.

Our differential MZI scheme measures the acceleration gradient between two spatially separated MZIs. The spatial separation $\vec{d}$ defines the direction of the accessed acceleration gradient, i.e., the magnetic field curvature $\vec{\nabla} B'_{z'} = (\partial_x B'_{z'}, \partial_y B'_{z'}, \partial_z B'_{z'})$ is measured along the spatial separation resulting in the quantity $\vec{d} \cdot \vec{\nabla} B'_{z'}$. For us, the spatial separation is in Bragg beam direction $\vec{d} = \vec{e}_{z'}$ such that we measure $\partial_{z'} B'_{z'} = \partial^2_{z'} |\vec{B}|$. In order to access other curvature components such as $\partial_x B'_{z'}$, one requires a spatial separation of the MZIs along $\vec{e}_x$ possible through alternative preparation schemes such as the one described in ref. [6].

In order to clarify this limitation as well as possible extensions, we added some discussion in the Results sections *II.B* and *II.C*.

Reviewer Comment 1.7 — While the idea of going to a zero-g environment is that perturbations go down and coherence times up, achieving this goal is still challenging. On the ground, on can have more atoms, better shielding, better vibration isolation, longer coherence time (than so far in space). In the outlook it would be interesting to see a 2-4 sentence timeline how the obstacles (everybody understands that they must be there at the start) can be overcome. This must be presented to NASA anyway.

Reply: We fully agree that the discussion of current performance limitations and how they could be overcome in future missions is vital. We already discussed some of these in our Discussion section in the second to last paragraph. To clarify the intention of this paragraph and to make it more executable for space agencies and those responsible for future missions, we restructured it. It is now clearly targeted at providing a recipe to improve sensitivities and fully exploit microgravity conditions in future space-based experiments. We additionally added suggestions for how to increase atom numbers and to add vibration isolation (reduce laser phase noise and compensate residual signals coupling in through the retro-reflection mirror).

Reviewer Comment 1.8 — The field curvature is given as $|B''| = (622.08 \pm 19.66)\,\mathrm{nT\,mm^{-2}}$. Given the error bar, it is probably more adequate to write this as $(620 \pm 20)\,\mathrm{nT\,mm^{-2}}$.

Reply: We rewrote the magnetic field curvature in line with the suggestion to $|B''| = (622 \pm 20)\,\mathrm{nT\,mm^{-2}}$. Additionally, we adapted $|B''| = (614.05 \pm 0.31)\,\mathrm{nT\,mm^{-2}}$ to $|B''| = (614.1 \pm 0.3)\,\mathrm{nT\,mm^{-2}}$.

Reviewer Comment 1.9 — It would be helpful to have a comparison: the best ground based optically pumped magnetometers can measure fT fields, but certainly not two in 1 mm distance. But how does all that compare and what is the cause for the curvature. What would be its time dependence? How much of that is locally generated by the atom chip ? How much is ISS noise?

Reply: We thank the reviewer for these relevant remarks. In accordance with the request, we now provide quantitative comparison between the sensitivity of an optically pumped scalar magnetometer with the projected sensitivity of future differential atom interferometers. As a basis for this comparison, we refer to the performance of the scalar magnetometer onboard the SWARM satellite mission [7] which measures the absolute magnetic field at a sensitivity of $1\,\mathrm{pT}/\sqrt{\mathrm{Hz}}$. As in this work, we are measuring the second order magnetic field gradient, we assume a three sensor array with distances of $40\,\mathrm{cm}$, analogous to the usual positioning of multiple fluxgates in traditional space magnetometry missions [8]. For this configuration, we derive a sensitivity of $15\,\mathrm{pT}/\mathrm{m}^2/\sqrt{\mathrm{Hz}}$ for traditional optically pumped magnetometers.

In total, we now provide several performance numbers for a differential atom interferometer that the reader can compare to. First, in section II.C, for an interferometer time of $2T = 10.3\,\mathrm{ms}$, we report our achieved sensitivity of about $5\,\mathrm{mT}\,\mathrm{m}^{-2}$ averaged over 181 shots. The parameters from this experiment correspond to a shot-noise limit of $0.4\,\mathrm{mT}\,\mathrm{m}^{-2}$. Additionally, we provide two achievable sensitivities for extrapolated performance parameters in future satellite missions. First, we evaluate the shot-noise limit for a differential atom interferometer with performance parameters envisioned for future fundamental physics tests [9] to $0.06\,\mathrm{pT}/\mathrm{m}^2/\sqrt{\mathrm{Hz}}$ @ $\lesssim 0.1\,\mathrm{Hz}$. For readers with particular interest for measuring signals at larger frequencies with more conservative parameters of 10^5 atoms per interferometer and a separation time of $T = 250\,\mathrm{ms}$, we evaluate the achievable sensitivity to $180\,\mathrm{pT}/\mathrm{m}^2/\sqrt{\mathrm{Hz}}$ @ $1\,\mathrm{Hz}$. Additionally, in the *Introduction* and *Discussion* sections, we now mention potential usage of our quantum sensor in a multi-modal way, allowing for simultaneous measurement of gravity gradients using the same scheme presented in this work.

For the measurement of magnetic field curvatures at distinct positions in the vacuum chamber of an atom interferometry experiment, we now also provide a more detailed motivation in the *Introduction* section, highlighting exactly the advantage of our method compared to using optically pumped scalar magnetometers outside of the vacuum chamber.

Regarding the cause of the curvature, in our *Discussion* section in lines 223-225, we mention *that the residual magnetic fields present within the CAL device [. . .] can be best explained by magnetic fields originating from the atom-chip connectors or other magnetized parts of the experimental setup*. We now additionally make the clarification that we expect these mentioned magnetized parts to be *inside the magnetic shield* to differentiate it from other magnetized parts in the ISS which CAL's magnetic shield is expected to isolate the measurement area from.

Apart from the statement in section II.C in lines 210 and 211 where *we conclude that actual drifts in the magnetic field curvature are the most likely explanation for the observed data spread*, we unfortunately do not know the time dependence of the magnetic field curvature. The main signal seems to be stable on a rather long time frame of several months, since the measurements for different interferometer times were also spread over such times and are consistent with each other. Nevertheless, smaller changes can occur on the order of seconds to hours or weeks, which was not possible for us to test due to CAL's mode of operation which usually does not allow for continuous operation without interruptions.

Reviewer Comment 1.10 — And why is B curvature more relevant than B or grad B ? Do they get these numbers also and if so with which accuracy ? Normally this is what the standard metrologist would search for. Being capable of seeing the curvature is a great thing. But again: start from a common ground.

Reply: We thank the reviewer for pointing out this critical point. Indeed, this work focuses on the measurement of magnetic field curvatures, mainly driven by the exploited differential atom interferometer

geometry. While a non-differential interferometer would measure the first order gradient of the potential experienced by the atoms, our differential scheme measures the second order potential gradient. In our case, this corresponds to a magnetic field curvature. To better guide the reader in understanding where the curvature comes in, we added a statement in the *Introduction* in line 62 that the differential scheme suppresses laser phase noise, *while directly measuring second order potential gradients (magnetic field curvature) where a non-differential scheme would measure the first order.*

Of course, we do not claim that measurements of magnetic field curvatures are more relevant than magnetic field gradients or their absolute values. There are three main reasons why scientifically we find it exciting to perform these measurements: first, it provides a relevant showcase for a space-deployed quantum sensing campaign of an external force via BEC interferometry, second, it will be crucial for in-vacuum characterization of higher order magnetic field gradients in atom interferometry based fundamental physics experiments and, third, the same interferometry scheme can be deployed for studying gravity gradients. We adapted the *Introduction* to more clearly state the second and third motivation for the study of magnetic field curvatures in our work by clearly highlighting two different applications of our magnetic field sensor in space in two separate paragraphs, *targeting the in-vacuum characterization of magnetic fields in aforementioned fundamental physics experiments based on atom interferometry* and *as part of a multi-modal quantum sensor for various low-frequency geophysical parameters*.

To clarify the constraints of our experimental setup, we now also added a paragraph to the *Magnetometry* section II.C where we discuss the different orders of magnetic fields and corresponding achievable accuracies. This also includes a short statement about the measurement of the absolute magnetic field value, which is possible with a differential atom interferometer, however it relies on a different idea of performing a differential measurement between different magnetic states rather than spatially separated states [10].

Reviewer Comment 1.11 — The ellipse fitting is a very useful tool in a noisy interferometer environment and I understand that the authors feel this should by now be known by everyone. But it is not. So before talking about bootstrapping algorithms (which I could not follow in the main text) they should remind us (evtl. also with a reference) why this helps seeing contrast even when a single interferometer alone is dephased. NCOMMS is also read by chemists and geodesist.

Reply: We thank the reviewer for pointing this out. To improve the manuscript, we have restructured the way we introduce the idea of ellipse fitting and now discuss the different shapes that the correlation data can have as well as the fact that they are not changed by common phase contributions in the main text. The technical details of the ellipse fitting process itself have been moved to the Methods section including the error estimation through bootstrapping methods.

Reviewer Comment 1.12 — The system is currently still limited by atom number (3×10^3 atoms per exit port) which constrains sensitivity and noise. What are the prospects for eventually pushing this by 3 orders of magnitude?

Reply: Indeed, future space-based atom interferometry experiments are planning to increase the number of atoms in the BEC to about 10^6, e.g., the CAL successor BECCAL [11] as well as the proposed space mission STE-QUEST for testing general relativity [9]. In the current instrument, the limiting factor why we cannot achieve larger atom numbers is the loading of thermal atoms from the MOT to the pure magnetic trap created by the atom chip. Here, the mode matching in terms of magnetic trap size,

position and frequencies is non-ideal. Therefore, for the future CAL science module, JPL decided to add mesoscopic wires below the two atom chip layers. Such a configuration has been used, for instance, in the MAIUS sounding rocket BEC machine [12, 13] as well as in the QUANTUS experiment [14]. A recent publication presenting a dual-species BEC machine for microgravity research realized more than 1.5×10^5 atoms in a Rb BEC [15], suggesting that reaching the numerously proposed 10^6 atoms in the BEC will require only incremental adaptions compared to current instruments. We added a sentence regarding this limitation to our *Discussion* section.

Being able to push the number of atoms by about three orders of magnitude will result in an improved shot noise limit for the sensitivity by a factor of $\sqrt{N_{\text{factor}}} = \sqrt{10^3}$. As mentioned in the reply to comment Comment 1.9, we now added two sensitivity projections for future space missions in the *Discussion* section. The first example is based on the performance parameters of the STE-QUEST mission [9] using $N = 10^6$ atoms in total. This results in a magnetic field curvature sensitivity of $0.06\,\mathrm{pT/m^2/\sqrt{Hz}}$ @ $\lesssim 0.1\,\mathrm{Hz}$. Additionally, we provide a second example targeted at readers with particular interest for measuring signals at larger frequencies with more conservative parameters of 10^5 atoms per interferometer. Using a smaller separation time of $T = 250\,\mathrm{ms}$, we evaluate the achievable sensitivity to $180\,\mathrm{pT/m^2/\sqrt{Hz}}$ @ $1\,\mathrm{Hz}$.

Reviewer Comment 1.13 — The achieved sensitivity still needs a factor of 10x to get to the shot-noise limit. So even without growing the number of atoms in the BEC: what can be done ?

Reply: We underline the importance of this observation and believe there are two crucial improvements that need to be addressed.

First, the cause for not reaching the shot-noise limit should be mitigated. These are, as we mention in lines 200 and 201, most likely "signal and experiment drifts in between shots, noisy imaging at small atom numbers, and consequently non-ideal ellipse fitting performance". Signal drifts in between shots constitute a real limitation to maintaining sensitivity at the shot noise limit over extended campaign durations, and for signals of interest with high frequency changes, this can be the sensitivity limiting factor. Experimental drifts on the other side can be mitigated, e.g., by improved temperature stabilization and operation at a stable instrument state in equilibrium, meaning both macroscopic hardware components as well as reducing CoM and size expansion instabilities in the BEC source preparation. While here it was asked for what can be done without growing atom numbers, non-ideal ellipse fitting performance is an artifact of small atom numbers. Reaching a certain threshold for the atom numbers would solve this problem as it will mitigate noisy signals where there is near-zero atom population in one of the ports and remove this limitation for reaching the shot noise limit for whatever atom number larger than this threshold.

Secondly, while it is included in the shot-noise limit, without increasing the number of atoms, a crucially improved sensitivity could be achieved with larger contrast. As detailed in the manuscript, this can be achieved via better and consistent pulse efficiencies, supported by a CoM velocity more aligned with the Bragg beam, a bigger, more homogeneous and higher power Bragg beam, as well as smaller BEC expansion velocities, both along and perpendicular to the beam. We expanded on some of these steps in the revised *Discussion*.

Reviewer 2

We thank the reviewer for their valuable feedback to our manuscript as well as their positive assessment of our pivot in the scientific pitch towards magnetic forces. Indeed, we are especially thankful for their suggestion in Comment 2.1 to add a quantitative comparison of our method to non-LPAI (light pulse atom interferometry) platforms. This helped us to critically reflect on feasible applications of our space-deployed differential atom interferometer, resulting in the proposal of integrating our magnetometer as a part of a multi-modal quantum sensor for various low-frequency geophysical parameters. We believe, this clarification of two separate future applications as well as the quantitative evaluation of future mission scenarios substantially improve the value of our paper.

In the following, we provide a point-by-point response to the reviewer's comments.

Reviewer Comment 2.1 — Based on the information contained in the manuscript, this work doesn't appear to be a sufficiently big technical or scientific leap compared to the previous two publications Ref. [46] and Ref. [45] by CAL in Nature and Nature Communications respectively. No hardware upgrades were required. The changes in the interferometer geometries appear to be the result of straightforward reprogramming. The scientific pitch is a bit stronger. As evident from the manuscript title, this work's scientific motivation presents a pivot from Ref. [45], which focused its science motivation on gravitational physics. This manuscript's introduction provides a strong motivation (e.g. geophysics) for in-orbit magnetic field sensing. Moreover, the authors adequately justify the advantage of localization of magnetic field measurements compared to equivalent Earth-based LPAI sensors. However, I am skeptical about LPAI being the most appealing platform for space-based magnetometry. Given the diverse readership of Nature Communications, the manuscript would more effectively sell the LPAI as a magnetic field sensor if *quantitative* comparison to non-LPAI platforms were presented. Even if current specs are not competitive with non-LPAI platforms, I recommend arguing a niche that only LPAI magnetometers can fill in future missions. My recommendation to publish in Nature Communications is contingent upon satisfactory responses to the above points.

Reply:

We thank the reviewer for rightfully raising the concern that our intended scientific motivations were not clearly presented, especially regarding the application for the measurement of geophysical parameters including a quantitative comparison with non-LPAI platforms. We sincerely revisited the science case, resulting in major adaptions of our *Introduction* and *Discussion* sections.

We see two scientific motivations for our demonstration of a magnetic field sensing campaign via differential atom interferometry in space. The first one concerns the in-vacuum characterization of magnetic fields in high precision fundamental physics experiments based on atom interferometry, for instance in STE-QUEST [9]. In such experiments, magnetic field gradients are one of the leading systematic effects coupling into the signal [16], and accurately mapping out these gradients is indispensable. To highlight this first motivation, we reworked the *Introduction* to now clearly separate it from the second motivation. Please refer to the adapted manuscript for further details.

Following the reviewers comment, we believe that especially our second scientific motivation required clarification. As highlighted by the reviewer, this second motivation concerns the in-orbit sensing of

geophysical parameters. Among such geophysical parameters are of course magnetic fields, where the presented sensing campaign in particular demonstrates the capability of measuring magnetic field curvatures. In addition to magnetic forces, it is of crucial importance that the same measurement scheme applied in this work can also be used to measure another important geophysical parameter, namely gravity gradients. Following the reviewers advise, we added a dedicated paragraph to outline the clear application case of a space-deployed multi-modal quantum sensor with the capability of measuring both gravitational and magnetic gradients. One of the key advantages of our sensor compared to traditional sensors is exactly this versatility in measuring different forces (and gradient orders thereof) using the same hardware via adaptions of the atom interferometric measurement schemes. In the following, we provide a quantitative comparison to non-LPAI platforms, on which outcome we fully based our above described suggestion on.

Before diving into this quantitative comparison, we need to establish the class of non-LPAI magnetometers to compare to. In the previous version of our manuscript, we mentioned SQUIDs as one class of high-precision magnetometers. While these are indeed among the most precise sensors in lab based setups, due to their requirement for cryogenics, these are not commonly used in space-based magnetometry missions. Rather two other kinds of magnetic field sensors are deployed in such missions, namely scalar atomic magnetometers [17] if one is interested in drift-free measurements of the absolute magnetic field and fluxgate sensors [18] for measuring the vector components of the magnetic field. Often, these devices are deployed in a hybrid system where the scalar atomic magnetometers are used to drift-stabilize the fluxgate sensors [7, 8, 19, 20]. Please note also our response to Comment 2.7 regarding the replacement of one of our references in accordance with our choice comparing to scalar atomic magnetometers and fluxgate sensors as non-LPAI magnetic field sensors.

To compare the sensitivity of these non-LPAI magnetometers to the ones achievable with differential atom interferometry, we need to investigate a configuration in which these sensors do not only provide (scalar or vector) values of the magnetic field itself but insight into their second derivative, i.e., the magnetic field curvature. We base our calculations on a configuration planned for a magnetometer for geomagnetic exploration onboard a satellite [8] which uses two fluxgates at a distance of $40\,\mathrm{cm}$. To measure the magnetic field curvature, we assume three scalar atomic magnetometers (instead of fluxgates, as the scalar ones measure the same quantity as our atom interferometer while performing at a better sensitivity than fluxgates) placed at a distance of $\Delta z = 40\,\mathrm{cm}$, respectively. Each measures the absolute magnetic field B at a sensitivity of $\sigma_B = 1\,\mathrm{pT}/\sqrt{\mathrm{Hz}}$ [7]. We calculate the curvature by taking the finite second order derivative via $B'' = \frac{B_1 - 2B_2 + B_3}{(\Delta z)^2}$ and obtain a propagated uncertainty of $\sigma(B'') = \frac{\sqrt{\sigma_B^2 + 2^2\sigma_B^2 + \sigma_B^2}}{(\Delta z)^2} = \frac{\sqrt{6}\sigma_B}{(\Delta z)^2}$. This results in a sensitivity of the scalar atomic magnetometers towards magnetic field curvatures of $15\,\mathrm{pT}/\mathrm{m}^2/\sqrt{\mathrm{Hz}}$.

We then compare this to the performance of shot-noise limited differential interferometers in dedicated future space missions. The shot-noise of a differential interferometer in the standard quantum limit is given by the propagated uncertainty from two Mach-Zehnder configurations. Each of those has a sensitivity to accelerations of $\sigma(a) = \frac{1}{k_{\mathsf{eff}} C \sqrt{N} T^2}$. By operating two Mach-Zehnder interferometers at a distance of $\Delta z = 40\,\mathrm{cm}$, the propagated uncertainty for the finite derivative is $\sigma(a') = \frac{\sqrt{2}}{\Delta z k_{\mathsf{eff}} C \sqrt{N} T^2}$. Considering the linear Zeeman effect, we calculate the sensitivity for two categories of future atom interferometric space missions. For the first one, we assume the performance parameters of a space mission proposal for tests of general relativity, namely, STE-QUEST [9]. For a contrast $C = 1$, a total of 10^6 BEC atoms split into two interferometers to $N = 10^6/2$ and an interferometer time of $T = 25\,\mathrm{s}$, we obtain a per-shot sensitivity of $8\,\mathrm{fT\,m^{-2}}$. Expressed as an amplitude spectral density with a cycle

time of $60\,\mathrm{s}$, the sensitivity evaluates to $0.06\,\mathrm{pT/m^2/\sqrt{Hz}}$ @ $\lesssim 0.1\,\mathrm{Hz}$. In order to provide a more near-term estimation of achievable sensitivities, we additionally consider more conservative parameters that could be realized in a dedicated mission with potential tradeoffs for higher versatility or operation with smaller cycle time. Here, $C = 1$, $N = 10^5$, a shorter interferometer time $T = 250\,\mathrm{ms}$ and a cycle time of $1\,\mathrm{s}$ result in a sensitivity of $180\,\mathrm{pT/m^2/\sqrt{Hz}}$ @ $1\,\mathrm{Hz}$.

We are convinced, such a LPAI-based multi modal sensor for measuring different order gradients of gravitational as well as magnetic forces will be compelling for the diverse readership of Nature Communications. To summarize, following the reviewer's advise, we made two major adaptions to our manuscript: 1) we reworked the *Introduction* to clearly and separately emphasize the two scientific motivations for the presented space-based magnetic sensing campaign and 2) added a thorough quantitative analysis of achievable sensitivities with LPAI-based sensors as part of dedicated missions compared to non-LPAI platforms.

Reviewer Comment 2.2 — Irrespective of the verdict of this submission, I think this work is of value to the microgravity LPAI community and might inform future missions like "BEC and CAL" (BECCAL). Hence, I believe publication (irrespective of location) is warranted after some revisions. In the following, I will identify possible avenues for strengthening the manuscript, elaborate on concerns about some of the measurements and list technical questions/comments which might be of relevance for future work.

1. In the conclusions, the authors' comment about Delta-Kick Cooling (DKC) being unnecessary (lines 260-262) appears to contradict an earlier statement (lines 147-148) that cloud expansion is one of the two factors limiting the visibility. Please reconcile those statements in the revised manuscript.

Reply: We thank the reviewer for catching the potential confusion arising from these two statements. Indeed, we did not clearly categorize the consequences of the two different effects of the atom's expansion velocity orthogonal to the Bragg beam and their orthogonal COM motion relative to the beam. In our experimental campaign, the more relevant factor is the atom's COM motion orthogonal to the beam during the interferometer. Due to this effect, for different interferometer times T, the Bragg pulses happen at different positions in the (diffracted) Gaussian beam profile. Due to limited experimental time on CAL as a multi-user facility, we could not take the time to measure and optimize the pulse durations for each of the positions in the beam (i.e., for each T). Thus, for longer (non-optimized) interferometer times we experience non-ideal pulse durations, eventually leading to a loss of contrast. As the $m_F = 0$ atoms are not (significantly) accelerated by magnetic field gradients, their transversal movement is reduced and we were able to obtain reasonable contrast at longer times.

In addition to the transversal COM effect, there is also a size expansion in the transversal directions. However, as the $m_F = 0$ allow for larger contrasts at longer interferometry times while their measured size dynamics are not significantly different, we do not believe this constitutes the leading limitation or would have significantly increased the accessible interferometer times. The size expansion, i.e., the momentum width in beam direction generally affects the achievable transfer efficiencies. However, we obtained transfer efficiencies of 85% with our source, confirming that also this was not the driving factor for our loss of contrast down to $< 25\%$.

One final reason why we did not implement DKC during this campaign was the already small number of atoms. As we discuss in different parts of the paper, this was indeed one of our main limitations. Performing an additional source preparation step in form of a delta kick would have only reduced the number of atoms further, amplifying these problems (without significantly improving the contrast for

the reasons discussed above). Future experiments that resolve the mentioned main limitations should of course implement DKC.

In order to clarify the above points, in the *Results* section II.B, we removed the mentioning of transverse expansion when explaining the limitation in interferometry time. Additionally, in our *Discussion* section, we now more clearly state that we regarded DKC as unnecessary during our campaign as it did not constitute the limiting factor and highlight DKC as a logical next step for improving transfer efficiency once the atom's COM motion has been eradicated as a limiting factor. To complement the reader's understanding of the atom's COM and size dynamics, we also adapted Fig. 1 to provide insight into these quantities.

Reviewer Comment 2.3 — On a related note, is DKC possible with the redesigned atom chip of SM-3? I didn't find any mentions of DKC capability in Refs. [45,46]. Moreover, in the methods section of Ref. [47], it says: "Later on, the core part of the apparatus, including the atom chip, was exchanged leading to a new magnetic field geometry and thus preventing the further continuation of the experiments reported in this article." Was this because the Z-, H-, U-traces were moved further apart to accommodate the Bragg beam? I leave it optional to elaborate on this in the manuscript if the authors are struggling with space constraints. Though, I encourage the authors to include these details in other publications.

Reply: Generally, DKC would be possible with the SM3 atom chip design, the requirements to the magnetic trap properties (trap frequencies, switching behaviour) for a similar scheme as reported in [34] (we believe accidentally referred to as [47] in the above comment) are fulfilled with the available chip geometries and current drivers. In [34], the continuation was prevented due to the inconsistency in trap geometries and hardware change rather than a general loss of DKC capabilities.

In this work, in order to lift the degeneracy of $\pm 2\hbar k$ in the Bragg transition, at the end of the BEC evaporation and transport in the magnetic trap, we perform a release scheme (as detailed in Chapter II.A) resulting in a finite velocity along the Bragg beam. This could in principle be realised in combination with a DKC scheme, either by applying an external magnetic field gradient in beam direction after the delta kick (which the SM3 hardware does not support) or by engineering a dedicated scheme with optimised release velocities out of the delta kick trap. The latter comes with a significant effort and requires investigation in a dedicated study, and with a focus of the present study lying in the demonstration of magnetic field sensing capabilities via atom interferometry and due to limited experiment time on this multi-user facility, we refrained from pursuing DKC here.

Please also note our explanation regarding the necessity for DKC within our campaign in our response to Comment 2.2.

Reviewer Comment 2.4 — 2. The manuscript highlights limitations on 2T to 24.3 ms for the $m_F = 2$ interferometers (lines 147-148 & 164-166) arising from transverse COM motion of the atom cloud (and cloud expansion) relative to the Bragg beam center due to accelerations from residual magnetic field gradients. BECCAL is supposed to have a piezo actuated tip-tilt mirror for rotation compensation. How much visibility improvement can be obtained by utilizing this feature to center the beam on the atom cloud? Alternatively, could the transverse atom cloud motion be converted from a bug to a feature by using the control signals from the rotation compensation system to tease out transverse magnetic fields?

Reply: In order to evaluate this idea, we shall recall the reasons why the transverse COM motion leads to reduced contrast. For all $m_F = 2$ interferometer times T, identical pulse times have been

employed, only the detuning has been adjusted individually. Thus, due to the small beam diameter as well as optical diffraction influencing its intensity distribution, the transversal CoM motion leads to different Bragg transition efficiencies for the outer $\pi/2$-pulses, varying with interferometer time T. The best solution to increase contrast would be to employ larger beam waists as well as to avoid optical diffraction of the beam, both leading to a more constant pulse intensity over different CoM positions as well as cloud sizes. Partially better pulse efficiency, and thus contrast improvement, in our setup could have been achieved by optimizing pulse durations individually for each interferometer time T. Due to limited experiment time on this multi-user facility, this was challenging to realize. In the future, using the actuated tip-tilt mirror requires a beam size large enough such that an overlap is maintained between the incident and (with an angle) reflected beam. However, this strategy cannot be used to mitigate the atoms (potentially) leaving the beam as one would need to change the position of both, incident and reflected beam, the BECCAL tip-tilt mirror however only adapts the reflected one. Indeed, this is also the common limitation for tip-tilt mirror setups when canceling the effect of rotations. We added a statement in the Discussion section regarding the need for tip-tilt mirrors to deal with platform rotations at significantly larger times and in non-differential setups. Please also refer to our reply to Comment 1.5 for a discussion of our sensitivity to rotations.

Reviewer Comment 2.5 — 3. Aside from rotation compensation, could visibility be improved by using quantum optimal control? I understand that CAL's current hardware has a limit on time resolution ($\sim 10\,\mu s$) for amplitude control of atom optics pulses. So, generating non-square pulse shapes might be challenging. What is the temporal phase resolution? Decent phase control would permit adiabatic rapid passage (ARP) on the Bragg beams. I only found discussion of ARP in the context of radio frequency transfer between $m_F = 0$ and 2. There are many examples in the literature improving pulse transfer efficiency via phase-only quantum control. Could the authors exploit such schemes to improve pulse transfer efficiency beyond the current 85%?

Reply: Indeed, quantum optimal control, in particular, an adiabatic-rapid-passage Bragg transition might be helpful to mitigate the effects of varying beam intensity over the atomic cloud and finite momentum width on pulse efficiency. In the present CAL module SM3, the control over the laser phase is limited. The Bragg pulses are generated by an AOM with the frequencies provided by an AWG (part number PXI-5442), limiting the waveform control to $5\,\mathrm{ns}$. While the CAL control software is configured to allow phase chirps in steps of $10\,\mu s$ in a single-tone scheme, for the dual-tone scheme required for the moving Bragg lattice, the phase control is limited to staircase steps of $10\,\mu s$ by the general CAL timing system. This time resolution allowing for only 20 steps during our mirror Bragg pulse with a duration of $200\,\mu s$ might restrict the achievable improvement in pulse efficiency beyond 85%. However, the capability to program in linear phase chirps for the multi-tone scheme might be possible to include in a future CAL upgrade. Indeed, we will seriously explore this option and thank the reviewer for this suggestion. We added a statement in the Methods section regarding the potential of using quantum optimal control and the limitation due to the current instrument software.

Reviewer Comment 2.6 — 4. With the information provided in the manuscript, empirical support for judging the veracity of the BFI results is inadequate. This is especially important given that the conclusion is a null result on the third spatial derivative of the magnetic field. This is in stark contrast to the differential MZI which is corroborated with an independent non-interferometric differential COM motion measurement of the atom clouds. As a proof-of-principle, can the authors inject a fake third spatial derivative of the magnetic field signal to build confidence in the differential

BFI? If the current hardware doesn't support injection or the mission schedule is too restrictive, I recommend including as much additional theoretical support and/or additional data as possible for the interpretation of the differential BFI results. For example, a skeptical reader would benefit from augmenting Fig. 3d to include visibility data from BFI-I and BFI-II.

Reply: We thank the reviewer for their suggestions. Unfortunately, there is no way of performing additional measurements with the setup that was used for the presented results as it got changed in the meantime. Injecting a fake signal with the same setup is therefore not possible, but it would be interesting to test this in a future mission.

We agree that the amount of supporting data for the BFI was not on the same level as for the differential MZI campaign which we have changed in the revised version of the manuscript. For this, we added a subplot to Figure 4 that shows the visibilities for all three campaigns as a function of interferometer time. Similar to the MZI with $m_F = 2$ atoms the visibilities decrease for growing interferometer time. To further support the BFI results, we also show the correlation data for the three different interferometer times in a new supplementary figure 8. Clearly, the three ellipses are very elongated and are consistent with a vanishing differential phase. For completeness we also added similar data for the differential MZI campaign with magnetic insensitive $m_F = 0$ atoms.

Moreover, in the BFI case we can also use the non-interferometric differential COM motion to show that they are consistent with each other. The distance between the two momentum states shown in Figure 7 is fitted with a sine function (Eq. 3) that is the exact solution for quadratic potentials. The fit shows an extremely high fidelity ($R^2 = 0.9999$) such that there is no indication of additional potential contributions to the dynamics. Hence, if there would be relevant higher magnetic field gradients, this would result in different behavior of the COM motion. Therefore, we are confident that the differential BFI shows a null result for third spatial derivatives of the magnetic field up to the achieved sensitivity. We have added a statement about this finding in the revised manuscript.

Reviewer Comment 2.7 — 5. Minor point: Ref. [36] is a review article on the superconducting gravimeter. Given this manuscript's focus on magnetism, it would make more sense to cite a standard reference on Superconducting Quantum Interference Device (SQUID) magnetometers.

Reply: We thank the reviewer for the level of detail they without doubt applied during their reviewing of this article, leading to pointing out this non-ideal citation [21]. Later during the rework of our manuscript, and especially when addressing their request for quantitative comparison of our magnetic field sensing performance to non-LPAI platforms, we realized that SQUIDs are generally not the best matching magnetic field sensor to compare to in our case. As we are proposing a magnetic field sensor on a satellite platform, we should rather refer to magnetometers that are typically deployed in such environments. Here, SQUIDs are usually avoided due to their challenging requirement for cryogenics.

Instead, we are now referring to two more relevant non-LPAI magnetometers, namely vector fluxgate magnetometers [18] and scalar atomic magnetometers [17] with the just mentioned general reviews. Such sensors are also commonly used in space magnetometry missions [8, 19, 22] where they use hybrid systems of vector fluxgates for performing the core measurements and scalar atomic magnetometers as absolute references for calibrating the drifting fluxgates.

We have therefore removed the citation ref. [21] altogether.

Reviewer 3

We thank the reviewer for accepting to referee our manuscript and describing our experiment as extremely impressive. However, we respectfully disagree with their conclusion regarding the impact of our work and believe they have not considered the challenging reality of deploying quantum-based technologies for metrology experiments in space. Below, we provide a more in-detail response to their general feedback as well as a point-by-point response to their specific comments.

Reviewer Comment 3.1 — The manuscript is clear, and technically sound. However I have some concerns about its novelty and impact. Several manuscripts were already published by the same group describing earlier results and establishing the basic operation of their system. Indeed the ability to carry such complicated and sensitive experiment in space is extremely impressive and does open exciting possibilities for future high impact experiments in 2 aspects: first measuring effects such as gravitational waves and planetary missions where space is inherently needed and second: micro gravity can significantly improve interrogation times (especially with the super cold atomic source they use) and also improve compactness and hence localization of the measurement. In the current manuscript the measurement is not important by itself but rather a demonstration and (partial) validation of another technical aspect: measurement the residual magnetic gradient that will be important for future precision measurement. Moreover the technique used is similar to previous atomic gradiometers demonstrated on earth. Finaly the quality of the measurement is not impressive on absolute scale (again the ability to carry such experiments on space is indeed impressive): interrogation times are rather small (probably due to the sub mm diameter of their laser beams where on earth people routinely use several cm beams and demonstrate 100 times longer interrogation). Also their signal to noise is quiet poor as compared to many earth experiments due to the small number of atoms they use.

To conclude: while in general the pursuit of atomic interferometers in space is highly motivated, advancing well and has many exciting prospects I find the current manuscript describing another technical step without strong innovation in methods or results and while clearly needed for future measurements is only marginally providing the wide impact needed for nature com.

Reply: We disagree with the reviewer's evaluation of our work as another technical step.

- Our campaign is the first that performs differential atom interferometry with BECs in orbit and proofs how to practically suppress omnipresent space-craft vibrations such that interference signals are turned into real measurements of physical quantities.
- Our butterfly geometry allows to access additional spatial derivatives of the magnetic field enabling a multi-mode operation being demonstrated for the first time in space.
- Our careful characterization of the Bragg beam and its environment significantly advances the understanding of remote operation of a multi-user platform and enables 10 fold longer interferometer times due to optimized atom source preparation and improved pulse efficiencies.

For these reasons, we are convinced our manuscript provides the required impact that the readership of *Nature Communications* is interested in. As articulated by the first reviewer, and seconded by the second reviewer, the present work "is an impressive technological feat and as such already interesting for the

global readership of Nature Communications". We are convinced that without publishing such milestones in high-impact journals such as *Nature Communications*, future upcoming missions will be severely impaired due to a lack of support by the surrounding science community as well as funding agencies. As the first reviewer states, our work "prepares and supports upcoming missions [...] and provides crucial lessons for planned fundamental physics experiments in space". In addition to these technological demonstrations, it is the second reviewer who highlights that "this manuscript's introduction provides a strong motivation (e.g. geophysics) for in-orbit magnetic field sensing", which is now supported with a quantitative comparison to traditional sensors, making it highly relevant for the diverse readership of *Nature Communications*.

It is crucial to note the challenging reality of deploying quantum sensors to space and advancing them from proof-of-principle experiments to metrologically relevant instruments. Our work reports on the first sensing campaign of an external force using a BEC interferometer. Thus, it clearly goes beyond the manuscripts mentioned by the reviewer that were mere demonstrations of coherence at some given interrogation time. As detailed in our Introduction and Discussion sections, we demonstrate the space-deployed capability of measuring different force gradients and orders with a single experimental setup while demonstrating the opportunities as well as limitations of differential atom interferometry, all of which are highly relevant for planning and executing various future space missions featuring atom interferometry.

Surprisingly, the reviewer recognizes some of our contributions by stating that "the ability to carry such complicated and sensitive experiment in space is extremely impressive and does open exciting possibilities for future high impact experiments" but seems to not acknowledge the fact that the presented experimental achievements cannot yet impress on absolute scales, given the fact they have been achieved in a multi-user facility with limited capabilities, making it even more important that these results are publicly recognized such that future generations of scientific programs and missions can build on top of these milestones. For example, we agree that future experiments need to work on increasing the interferometer times by one or two orders of magnitude more. However, we believe our demonstration of ten-fold interrogation times within a magnetometry sensing campaign compared to previous realizations needs to be recognized as such.

Finally, we would like to comment on the statement that 100 times longer interrogation times (this would be $2T \geq 4\,\mathrm{s}$) are routinely demonstrated on Earth. The reviewer definitely has a point here in that our achieved interrogation times are still smaller than many that have been realized on Earth. However, while still smaller, the key value of increasing the interrogation time in a space-deployed experiment by an order of magnitude is that these methods have the potential to scale up to tens of seconds [9] technical constraints (partially demonstrated in this work) are resolved. The order of a second interrogation times achieved on Earth are not sensible to scale much further due to the free fall distance scaling quadratically with time. This includes various platforms such as the $10\,\mathrm{m}$ atomic fountain achieving a total interrogation time of $\sim 2\,\mathrm{s}$ [16], the atom interferometry experiments in the $\sim 100\,\mathrm{m}$ Bremen droptower with achieved times on the order of $\gtrsim 0.5\,\mathrm{s}$ [23], ultimately limited to about $9\,\mathrm{s}$ or the realized interrogation time of $0.2\,\mathrm{s}$ in an Einstein Elevator [24] ultimately limited to $0.5\,\mathrm{s}$. Therefore, although not yet competitive in interrogation time, working on the required methods to push the interrogation times in space-deplyed experiments will open up scales not achievable on Earth.

Reviewer Comment 3.2 — Few technical comments (line numbers in brackets): 1. (62) "This allowed the execution of various differential interferometer geometries up to total interrogation times of 2T = 40.3ms..." considering this is important part of the paper novelty - there should be

an elaborate explanation of how they achieved this. As I understand it, differential interferometry isn't enough to overcome their previous limitation.

Reply: Indeed, the ability to increase the interrogation time of BEC interferometers in space to $2T = 40$ ms is an important result of our work and its novelty and goes beyond the results of previous space-based BEC interferometers by one order of magnitude. There are two distinct aspects that are important here and allowed us to perform these measurements on a new level: (i) getting the interferometer to work on these time scales in space to produce a signal with enough visibility and (ii) to extract the physical quantity of interest out of that signal.

The first key driver (i) to enable extended interferometer times is the ability to achieve a high visibility for the correlated data after long interferometer times. For this purpose our proper characterization of the Bragg beam properties as well as design of the atom source preparation and trap release scheme to ensure overlap of the Bragg beam with the atom cloud for as long as possible and thus efficient interferometer pulses was essential. Only in this way the interferometer signal at the end of the sequence has a relevant visibility and meaningful results can be extracted. Here, we have reached a new level of control and understanding of the apparatus compared to previous implementations with CAL. First, we performed a detailed characterization of the Bragg beam orientation and intensity in order to precisely know where its intensity is maximal. Based on this knowledge we optimized the atom source preparation and release scheme to be aligned with the Bragg beam as much as possible despite the omnipresent magnetic field gradients that accelerate the atoms away from the beam center. Finally, we also engineered the transport sequence towards slow expansion dynamics (see new Fig. 1d) featuring 0.66 nK effective temperature in the beam direction to support high pulse efficiencies. This detailed characterization and preparation campaign was the main driver that allowed us to improve the interferometer times and overcome previous limitations and thus sets this work apart from previous results obtained with CAL.

For the $m_F = 0$ atoms especially the alignment with the Bragg beam was easier to establish and to keep over longer expansion times such that these experiments allowed us the longest interferometer times reported here.

For the second part (ii), i.e., extracting the signal of interest, differential interferometry was vital because it allowed us to extract the differential phase out of the correlated signals of the two MZIs. While their individual phase scan results are both completely washed out due to phase noise from the ISS vibrations, the combined result allows the precise determination of a differential phase and thus measurement of acceleration gradients (magnetic field curvature in our case). Here our results are the first demonstrating such techniques in space with a BEC.

These aspects have been discussed in the main part of the paper already, and we have adjusted the statement made in the introduction to make these key points clearer. We also extended some of the explanations in the main part of the paper accordingly.

Reviewer Comment 3.3 — 2. (68-69) The bound on non-magnetic acceleration gradient is expressed strangely, it's sufficient to say that they've bound it below $0.3s^{-}2$. Honestly it's an insignificant result of this work and holds value merely as a sanity-check. I would omit it from the introduction entirely.

Reply: We adapted the statement regarding the non-magnetic acceleration measurement. We agree that the more relevant statement is that we performed a non-magnetic measurement at all, and that we generally put a constraint in the sense of a sanity-check. The sentence now reads as follows: "Deploying $m_F = 0$ atoms, we verify the linear Zeeman effect as the leading source of the measured curvature and provide an upper bound to other contributions."

Moreover, the $m_F = 0$ allowed us to go beyond the $m_F = 2$ interferometer times due to absence of magnetic field accelerations and thus better control over the COM motion and better alignment with the Bragg beam over longer times. The expansion rates of both campaigns are very similar since both use the same evaporation, transport and release proceedure. As a result the $m_F = 0$ measurement helped us to also verify the limitations of our implementation, which is important to go beyond this in the future.

Reviewer Comment 3.4 — 3. (111) It should be made clear that this acceleration gradient measurement is classical with no interferometry involved.

Reply: Indeed the acceleration gradient measurement through differential COM motion is classical with no interferometry involved and serves as an independent comparison. We adjusted the statement to make this fact clearer.

Reviewer Comment 3.5 — 4. (140) There's a phase ambiguity when the 2 interferometers are in-phase. A frequency shifted mirror pulse can add an artificial phase shift between the clouds and solve this issue (PRL 118, 160401 (2017)). If this solution is prohibitive in CAL, it's worthwhile to mention it.

Reply: This is certainly a good suggestion. One could indeed consider adding an artificial contribution to the differential phase shift through a jump of the detuning from the excited state of the Bragg pulses, as done in ref. [25]. However, in our case the separation between the two interferometers is about a hundred times smaller (0.3 mm compared to 3 cm), and the relevant interferometer times are shorter by a factor of 2 or more. The required frequency jump is inversely proportional to the separation and the square of the interferometer time, and it is therefore 400 times higher or more in our case, which amounts to a frequency shift of order 10^2 GHz and is not a viable option with our setup.

We have added an explanation of this point in the ellipse fitting section of the Methods in the revised manuscript.

Reviewer Comment 3.6 — 5. In general precise measurement of the in-chamber magnetic gradient isn't motivated well enough

Reply: We appreciate that the motivation for in-chamber magnetic gradient characterization felt a bit rushed. We reworked the paragraph to make clear that it is one of two main space applications we foresee for our magnetic field sensor. We also emphasized the generality of this application to all fundamental physics experiments based on atom interferometry, only mentioning tests of general relativity as an example. We strengthened the reasoning for why outside of vacuum chamber measurements are not sufficient for, and that the use of our method enables the characterization at different positions and of different orders with minor adaptions of the measurement scheme. Additionally, we argue that the in-orbit characterization of in-chamber magnetic gradients is advantageous as it provides more localized measurements to the absence of gravity.

Additional changes

We adjusted the wording and corrected minor errors throughout the manuscript.

References

[1] D. C. Aveline, J. R. Williams, E. R. Elliott, C. Dutenhoffer, J. R. Kellogg, J. M. Kohel, N. E. Lay, K. Oudrhiri, R. F. Shotwell, N. Yu, and R. J. Thompson, "Observation of Bose–Einstein condensates in an Earth-orbiting research lab," *Nature*, vol. 582, no. 7811, pp. 193–197, Jun. 2020.

[2] E. R. Elliott, D. C. Aveline, N. P. Bigelow, P. Boegel, S. Botsi, E. Charron, J. P. D'Incao, P. Engels, T. Estrampes, N. Gaaloul, J. R. Kellogg, J. M. Kohel, N. E. Lay, N. Lundblad, M. Meister, M. E. Mossman, G. Müller, H. Müller, K. Oudrhiri, L. E. Phillips, A. Pichery, E. M. Rasel, C. A. Sackett, M. Sbroscia, W. P. Schleich, R. J. Thompson, and J. R. Williams, "Quantum gas mixtures and dual-species atom interferometry in space," *Nature*, vol. 623, no. 7987, pp. 502–508, Nov. 2023.

[3] J. R. Williams, C. A. Sackett, H. Ahlers, D. C. Aveline, P. Boegel, S. Botsi, E. Charron, E. R. Elliott, N. Gaaloul, E. Giese, W. Herr, J. R. Kellogg, J. M. Kohel, N. E. Lay, M. Meister, G. Müller, H. Müller, K. Oudrhiri, L. Phillips, A. Pichery, E. M. Rasel, A. Roura, M. Sbroscia, W. P. Schleich, C. Schneider, C. Schubert, B. Sen, R. J. Thompson, and N. P. Bigelow, "Pathfinder experiments with atom interferometry in the Cold Atom Lab onboard the International Space Station," *Nature Communications*, vol. 15, no. 1, p. 6414, Aug. 2024.

[4] M.-K. Zhou, Z.-K. Hu, X.-C. Duan, B.-L. Sun, L.-L. Chen, Q.-Z. Zhang, and J. Luo, "Performance of a cold-atom gravimeter with an active vibration isolator," *Physical Review A*, vol. 86, no. 4, p. 043630, Oct. 2012.

[5] S.-Y. Lan, P.-C. Kuan, B. Estey, P. Haslinger, and H. Müller, "Influence of the coriolis force in atom interferometry," *Physical Review Letters*, vol. 108, no. 9, p. 090402, 2012.

[6] K. Stolzenberg, C. Struckmann, S. Bode, R. Li, A. Herbst, V. Vollenkemper, D. Thomas, A. Rajagopalan, E. Rasel, N. Gaaloul *et al.*, "Multi-axis inertial sensing with 2d matter-wave arrays," *Physical Review Letters*, vol. 134, no. 14, p. 143601, 2025.

[7] J.-M. Léger, T. Jager, F. Bertrand, G. Hulot, L. Brocco, P. Vigneron, X. Lalanne, A. Chulliat, and I. Fratter, "In-flight performance of the Absolute Scalar Magnetometer vector mode on board the Swarm satellites," *Earth, Planets and Space*, vol. 67, no. 1, p. 57, Dec. 2015.

[8] B. Cheng, B. Zhou, W. Magnes, R. Lammegger, and A. Pollinger, "High precision magnetometer for geomagnetic exploration onboard of the China Seismo-Electromagnetic Satellite," *Science China Technological Sciences*, vol. 61, no. 5, pp. 659–668, May 2018.

[9] H. Ahlers, L. Badurina, A. Bassi, B. Battelier, Q. Beaufils, K. Bongs, P. Bouyer, C. Braxmaier, O. Buchmueller, M. Carlesso, E. Charron, M. L. Chiofalo, R. Corgier, S. Donadi, F. Droz, R. Ecoffet, J. Ellis, F. Estève, N. Gaaloul, D. Gerardi, E. Giese, J. Grosse, A. Hees, T. Hensel, W. Herr, P. Jetzer, G. Kleinsteinberg, C. Klempt, S. Lecomte, L. Lopes, S. Loriani, G. Métris,

T. Martin, V. Martín, G. Müller, M. Nofrarias, F. P. D. Santos, E. M. Rasel, A. Robert, N. Saks, M. Salter, D. Schlippert, C. Schubert, T. Schuldt, C. F. Sopuerta, C. Struckmann, G. M. Tino, T. Valenzuela, W. von Klitzing, L. Wörner, P. Wolf, N. Yu, and M. Zelan, "STE-QUEST: Space time explorer and quantum equivalence principle space test," 2022.

[10] K. S. Hardman, P. J. Everitt, G. D. McDonald, P. Manju, P. . Wigley, M. A. Sooriyabandara, C. C. N. Kuhn, J. E. Debs, J. D. Close, and N. P. Robins, "Simultaneous precision gravimetry and magnetic gradiometry with a bose-einstein condensate: A high precision, quantum sensor," *Phys. Rev. Lett.*, vol. 117, no. 13, p. 138501, Sep. 2016.

[11] K. Frye, S. Abend, W. Bartosch, A. Bawamia, D. Becker, H. Blume, C. Braxmaier, S.-W. Chiow, M. A. Efremov, W. Ertmer, P. Fierlinger, T. Franz, N. Gaaloul, J. Grosse, C. Grzeschik, O. Hellmig, V. A. Henderson, W. Herr, U. Israelsson, J. Kohel, M. Krutzik, C. Kürbis, C. Lämmerzahl, M. List, D. Lüdtke, N. Lundblad, J. P. Marburger, M. Meister, M. Mihm, H. Müller, H. Müntinga, A. M. Nepal, T. Oberschulte, A. Papakonstantinou, J. Perovšek, A. Peters, A. Prat, E. M. Rasel, A. Roura, M. Sbroscia, W. P. Schleich, C. Schubert, S. T. Seidel, J. Sommer, C. Spindeldreier, D. Stamper-Kurn, B. K. Stuhl, M. Warner, T. Wendrich, A. Wenzlawski, A. Wicht, P. Windpassinger, N. Yu, and L. Wörner, "The bose-einstein condensate and cold atom laboratory," *EPJ Quantum Technology*, vol. 8, no. 1, p. 1, jan 2021.

[12] D. Becker, M. D. Lachmann, S. T. Seidel, H. Ahlers, A. N. Dinkelaker, J. Grosse, O. Hellmig, H. Müntinga, V. Schkolnik, T. Wendrich, A. Wenzlawski, B. Weps, R. Corgier, T. Franz, N. Gaaloul, W. Herr, D. Lüdtke, M. Popp, S. Amri, H. Duncker, M. Erbe, A. Kohfeldt, A. Kubelka-Lange, C. Braxmaier, E. Charron, W. Ertmer, M. Krutzik, C. Lämmerzahl, A. Peters, W. P. Schleich, K. Sengstock, R. Walser, A. Wicht, P. Windpassinger, and E. M. Rasel, "Space-borne bose–einstein condensation for precision interferometry," *Nature*, vol. 562, no. 7727, pp. 391–395, oct 2018.

[13] M. D. Lachmann, H. Ahlers, D. Becker, A. N. Dinkelaker, J. Grosse, O. Hellmig, H. Müntinga, V. Schkolnik, S. T. Seidel, T. Wendrich, A. Wenzlawski, B. Carrick, N. Gaaloul, D. Lüdtke, C. Braxmaier, W. Ertmer, M. Krutzik, C. Lämmerzahl, A. Peters, W. P. Schleich, K. Sengstock, A. Wicht, P. Windpassinger, and E. M. Rasel, "Ultracold atom interferometry in space," *Nature Communications*, vol. 12, no. 1, p. 1317, feb 2021.

[14] C. Deppner, W. Herr, M. Cornelius, P. Stromberger, T. Sternke, C. Grzeschik, A. Grote, J. Rudolph, S. Herrmann, M. Krutzik, A. Wenzlawski, R. Corgier, E. Charron, D. Guéry-Odelin, N. Gaaloul, C. Lämmerzahl, A. Peters, P. Windpassinger, and E. M. Rasel, "Collective-Mode Enhanced Matter-Wave Optics," *Phys. Rev. Lett.*, vol. 127, no. 10, p. 100401, Aug. 2021.

[15] B. Piest, J. Böhm, T. Estrampes, A. Pichery, P. Arciszewski, W. Bartosch, S. Boles, K. Döringshoff, M. Elsen, P. Guggilam *et al.*, "Apparatus for quantum-mixture research in microgravity," *arXiv preprint arXiv:2508.20820*, 2025.

[16] P. Asenbaum, C. Overstreet, M. Kim, J. Curti, and M. A. Kasevich, "Atom-interferometric test of the equivalence principle at the 10^{-12} level," *Phys. Rev. Lett.*, vol. 125, no. 19, p. 191101, 2020.

[17] Y. Lu, T. Zhao, W. Zhu, L. Liu, X. Zhuang, G. Fang, and X. Zhang, "Recent Progress of Atomic Magnetometers for Geomagnetic Applications," *Sensors*, vol. 23, no. 11, p. 5318, Jun. 2023.

[18] S. Wei, X. Liao, H. Zhang, J. Pang, and Y. Zhou, "Recent Progress of Fluxgate Magnetic Sensors: Basic Research and Application," *Sensors*, vol. 21, no. 4, p. 1500, Feb. 2021.

[19] J. Heppner, N. Ness, T. Shillman, and C. Scearce, "Magnetic field measurements with the explorer x satellite," *J. Phys. Soc. Japan*, vol. 17, no. Suppl A, 1961.

[20] M. H. Acuña, "Space-based magnetometers," *Review of Scientific Instruments*, vol. 73, no. 11, pp. 3717–3736, Nov. 2002.

[21] J. M. Goodkind, "The superconducting gravimeter," *Review of Scientific Instruments*, vol. 70, no. 11, pp. 4131–4152, Nov. 1999.

[22] C. Amtmann, A. Pollinger, M. Ellmeier, M. Dougherty, P. Brown, R. Lammegger, A. Betzler, M. Agú, C. Hagen, I. Jernej, J. Wilfinger, R. Baughen, A. Strickland, and W. Magnes, "Accuracy of the scalar magnetometer aboard ESA's JUICE mission," *Geoscientific Instrumentation, Methods and Data Systems*, vol. 13, no. 1, pp. 177–191, 2024.

[23] H. Müntinga, H. Ahlers, M. Krutzik, A. Wenzlawski, S. Arnold, D. Becker, K. Bongs, H. Dittus, H. Duncker, N. Gaaloul, C. Gherasim, E. Giese, C. Grzeschik, T. W. Hänsch, O. Hellmig, W. Herr, S. Herrmann, E. Kajari, S. Kleinert, C. Lämmerzahl, W. Lewoczko-Adamczyk, J. Malcolm, N. Meyer, R. Nolte, A. Peters, M. Popp, J. Reichel, A. Roura, J. Rudolph, M. Schiemangk, M. Schneider, S. T. Seidel, K. Sengstock, V. Tamma, T. Valenzuela, A. Vogel, R. Walser, T. Wendrich, P. Windpassinger, W. Zeller, T. van Zoest, W. Ertmer, W. P. Schleich, and E. M. Rasel, "Interferometry with Bose-Einstein Condensates in Microgravity," *Physical Review Letters*, vol. 110, no. 9, p. 093602, Feb. 2013.

[24] C. Pelluet, R. Arguel, M. Rabault, V. Jarlaud, C. Métayer, B. Barrett, P. Bouyer, and B. Battelier, "Atom interferometry in an Einstein Elevator," *Nature Communications*, vol. 16, no. 1, p. 4812, May 2025.

[25] R. P. del Aguila, T. Mazzoni, L. Hu, L. Salvi, G. M. Tino, and N. Poli, "Bragg gravity-gradiometer using the $^1S_0 - {}^3P_1$ intercombination transition of ^{88}Sr," *New J. Phys.*, vol. 20, no. 4, p. 043002, 2018.

Author response to referee comments (round no. 2)

Manuscript "Space magnetometry with a differential atom interferometer"

NCOMMS-25-82843-T

Reviewer 1

We thank the reviewer for their recommendation for publication in *Nature Communications* as well as their ongoing effort in helping us improve the manuscript further.

In the following, we provide a point-by-point response to the reviewer's comments.

Reviewer Comment 1.1 — Title: "Space magnetometry with a differential atom interferometer". This reads as if the atom interferometer measured the B-field distribution of space, like Voyager travelling through the solar system. However, the measurements rather concern the very local field which needs to be known for instance as a background for WEP measurements later. Probably more correct: "Magnetometry with a space-based differential atom interferometer"?

Reply: We agree and have adapted the title to "Magnetometry with a space-based differential atom interferometer" as suggested.

Reviewer Comment 1.2 — Punctuation and orthography:

- Line 53: Single Bragg → single Bragg ?
- Line 63: "contributions.." only one "."

But these things will be sorted out during the editing process, anyway.

Reply: We have corrected these errors.

Reviewer Comment 1.3 — Vibration unit "order of $10^{-9}\mathrm{g}^2/\mathrm{Hz}$" is technically correct, but may sound unusual for a general reader, since it gives the acceleration power spectral density, not an acceleration. I suggest to give the acceleration amplitude, in a frequency band of 1 Hz and some information about the spectral variation: is this the max value at about 10-100 Hz of noise, which is the most relevant range for the measurements?

Reply: We have adapted how we express the vibrations from a PSD to an ASD as suggested. Our main intention with stating the vibration ASD is to convey to the reader the rough level of present vibrations which limit the usability of non-differential interferometry schemes. To avoid an overload with technical details and as we suppress vibrations as common mode noise in our differential scheme anyway, we refrain from discussing the frequency ranges further and expect interested readers will refer to the reference.

Reviewer Comment 1.4 — Punchline of the introduction

a) The authors want to make a double statement: 1. The have improved numerous parts of the experiment and overcome technical limitations of previous experiments in CAL. 2. Based on

that they have performed in situ magnetometry and obtain interesting values on gradients and curvatures. This is somehow captured in line 49, but there is an opportunity to formulate things more positively, by deleting line 47 - 50 and simply continuing with the improvements described starting with line 51.

Reply: We have adapted the mentioned paragraphs according to the reviewer's suggestions while maintaining the important statement about the application of a BEC interferometer as a magnetic field sensor.

Reviewer Comment 1.5 — b) Their lines 64-68

We foresee two different applications of the magnetic curvature sensor in space, the first one targeting the in-vacuum characterization of magnetic fields in aforementioned fundamental physics experiments based on atom interferometry. In such experiments, for instance, when testing general relativity8–10, one of the leading systematic effects coupling into the signal are magnetic field gradients. Fully avoiding such couplings is not possible, neither by operating on a magnetically insensitive sub-level due to the non-linear Zeeman effect, nor by advanced compensation techniques39. Thus, magnetic field gradients need to be mapped microscopically at the measurement location, i.e., inside the vacuum chamber where only the atoms have direct access. Devices such as vector fluxgate magnetometers and scalar atomic magnetometers generally offer high precision, however, they have to measure from out-of-vacuum, naturally impeding the performance for gradient reconstruction at the relevant location, especially considering higher order spatial components. In contrast, utilizing a differential atom interferometric magnetometer enables precise in-vacuum characterization of magnetic field gradients at the right spot. Multiple orders can be mapped out by deploying the differential interferometer at various positions or by adapting the scheme to be sensitive to a different order, as presented in this work. Due to the absence of gravity, performing such characterizations in orbit instead of during an on-ground commissioning phase provides the advantage of more localized measurements under the true environmental conditions.

Could be simplified and presented more: We foresee two different applications of the magnetic curvature sensor in space: first the in-vacuum characterization of magnetic fields for tests of general relativity, where one of the leading systematic effects is due to magnetic field gradients. Thus, magnetic field gradients need to be mapped microscopically at the measurement location, i.e., inside the vacuum chamber where only the atoms have direct access. Fluxgate magnetometers and atomic magnetometers can offer higher precision, however, they measure from the outside, impeding the reconstruction of gradient and higher order derivatives of the magnetic field. A characterization in orbit is free of the influence of gravity, allowing for more localized measurements under the experimental conditions of future gravity experiments.

One may find some more paragraphs where the key message can be conveyed more clearly simply by reducing or eliminating parts of sentences and avoiding repetition.

Reply: We thank the reviewer for this detailed feedback. We adapted this paragraph during the first review round according to the other reviewers' feedback with the goal of expanding the motivation for the in-vacuum magnetic curvature application. We believe the current level of technical detail is crucial to understand the impact of our presented experiments.

Reviewer Comment 1.6 — We perform two types of differential atom interferometer geometries

Is it grammatically ok to "perform a geometry" ? or would you want to say: We have realized differential atom interferometry in two geometries.

Reply: We have adapted the statement and removed the term "geometry" such that the sentence now goes as: "We perform two types of differential atom interferometers to characterize the gradient and curvature . . . "

Reviewer Comment 1.7 — Tenses

The text occasionally change tenses between different paragraphs. It feels a bit uneven...but the editing team will go through grammar and style during the final process, anyway.

Reply: We have adapted multiple occasions in the discussion section where we used simple past to now use present perfect. This should now be consistent throughout the manuscript.

Reviewer 2

We thank the reviewer for their recommendation for publication in *Nature Communications* and would like to express our gratitude again for their valuable feedback prompting us to better contextualize our work within the landscape of space-based magnetometry.

Reviewer 3

We thank the reviewer for considering our point of view regarding the future prospects enabled by the contributions within our manuscript as well as their positive assessment of the changes we made in the first review round.
